# Bioactive Alpha-Pyrone and Phenolic Glucosides from the Marine-Derived *Metarhizium* sp. P2100

**DOI:** 10.3390/jof9010028

**Published:** 2022-12-23

**Authors:** Zhong-Lian Ma, Zhi-Pu Yu, Yao-Yao Zheng, Na Han, Ya-Hui Zhang, Shu-Yue Song, Jun-Qiu Mao, Jiao-Jiao Li, Guang-Shan Yao, Chang-Yun Wang

**Affiliations:** 1 Key Laboratory of Marine Drugs, The Ministry of Education of China, School of Medicine and Pharmacy, Ocean University of China, Qingdao 266003, China; 2 Fujian Key Laboratory on Conservation and Sustainable Utilization of Marine Biodiversity, Institute of Oceanography, Minjiang University, Fuzhou 350108, China; 3 Laboratory for Marine Drugs and Bioproducts, Qingdao National Laboratory for Marine Science and Technology, Qingdao 266237, China; 4 Institute of Evolution & Marine Biodiversity, Ocean University of China, Qingdao 266003, China

**Keywords:** marine-derived fungus, *Metarhizium*, glucoside, 4-*O*-methyl-*β*-D-glucose, *α*-amylase inhibitor

## Abstract

Glycoside compounds have attracted great interest due to their remarkable and multifarious bioactivities. In this study, four hitherto unknown 4-methoxy-*β*-D-glucosyl derivatives were obtained and identified from the marine-derived fungus *Metarhizium* sp. P2100, including three alpha-pyrone glycosides (**1**–**3**) and one phenolic glycoside (**4**). Their planar structures were elucidated by comprehensive spectroscopic analysis, including 1D/2D NMR and HRESIMS. The absolute configurations of **1**–**3** were determined by a single-crystal X-ray crystallographic experiment, a comparison of the experimental, and a calculated electronic circular dichroism (ECD) spectra, respectively. Compounds **2** and **3** are a pair of rare epimeric pyranoside glycosides at C-7 with a core of aglycone as 2*H*-pyrone. Compounds **1**–**4** exhibited weak anti-inflammatory activities. In particular, compounds **1**–**3** displayed inhibitory activities against *α*-amylase, showing a potential for the development of a new *α*-amylase inhibitor for controlling diabetes.

## 1. Introduction

Glycosylation represents one of the most common and essential biochemical reactions in vivo. The resulting glycoconjugates possess diverse functions, including information storage and transfer, energy storage, maintenance of cell structural integrity, molecular recognition, signaling, virulence, and chemical defense [1]. Glycosylated modification of natural and synthetic small-molecular drugs could significantly alter the pharmacological properties of parent compounds [2]. Glycoside compounds fall into several main structure types by aglycone, including pyranone, flavone, alkaloid, macrolide, iridoid, etc. A significant number of these molecules have been clinically used as antibiotics, enzyme inhibitors, hormones, and drugs for the treatment of human diseases [1]. For example, streptomycin, gentamycin, and vancomycin are typical representatives of glycosidic antibiotics that have been used as antibacterial agents [3]. Amphotericin B [4] and nystatin A1 [5] have been used as potent antifungal drugs. Acarbose has been used to treat diabetes as an alpha-glucosidase inhibitor [6]. Cardiac glycosides have been used to treat various heart conditions [7].

In recent decades, with the development of marine biological resources, marine organisms-sourced secondary metabolites have played an important role in the discovery and development of novel drugs [8,9,10]. Among these bioactive natural products, secondary metabolites derived from marine fungi account for an increasing proportion, and more and more bioactive glycosides have been excavated [11,12]. These include 3-*O*-(6-*O*-*α*-L-arabinopyranosyl)-*β*-D-gluco-pyranosyl-1,4-dimethoxyxanthone, and xanthone *O*-glycoside, obtained from the mangrove endophytic fungus *Phomopsis* sp. and found to display cytotoxicity against HEp-2 and HepG2 cells with IC_50_ values of 9 and 16 µM, respectively [13]. Aquastatin A was isolated from a marine-derived fungus *Cosmospora* sp., displaying potent and selective inhibitory activity against protein tyrosine phosphatase 1B (PTP1B) with an IC_50_ value of 0.19 µM in a competitive manner [14]. A series of virescenosides were obtained from *Acremonium striatisporum*, isolated from sea cucumber, among which virescenosides M–U displayed cytotoxic activity against the tumor cells, Ehrlich carcinoma, in vitro [15,16].

*Metarhizium* is a class of entomopathogenic filamentous fungi. It plays a vital role in the protection of the ecological environment and the development of green agriculture [17]. Its secondary metabolites perform many functions, such as mediating intra- and interspecies communication, and mitigating abiotic and biotic stresses in the process of insect infection [18]. There are abundant *Metarhizium* resources in nature, and novel *Metarhizium* species have been found continuously. It was reported that the genomic sequencing of existing *Metarhizium* revealed an abundance of secondary metabolite biosynthesis gene clusters with great potential in the mining of novel secondary metabolites [19]. Considering the diversity of the genome and secondary metabolome, it is expected that the structurally unique and bioactive compounds may be more prone to be identified in new species or strains than the known ones.

During our continuous research, aiming to explore bioactive natural products from the marine environment, a variety of new secondary metabolites with multiple biological activities have been obtained from marine microorganisms [20,21,22,23]. In the present study, the chemical investigation of the marine-derived fungal strain *Metarhizium* sp. P2100 was performed, which was isolated from the seawater of the Yellow Sea, China. Four new glucoside compounds were isolated and identified. Herein, we reported the isolation, structure elucidation, and biological activities of these compounds.

## 2. Materials and Methods

### 2.1. General Experimental Procedures

Optical rotations were measured on a JASCO P-1020 digital polarimeter (Jasco Corp., Tokyo, Japan). UV spectra were recorded on a HITACHI UH 5300 UV spectrophotometer (Hitachi, Tokyo, Japan). The ECD data were acquired on a Chirascan Circular Dichroism spectrometer (Applied Photophysics Ltd., Leatherhead, UK). The IR spectra were recorded on a Nicolet-Nexus-470 spectrometer (Thermo Electron Co., Madison, WI, USA) using KBr pellets. The NMR spectra were acquired by a Bruker AVANCE III (400 MHz for ^1^H and 100 MHz for ^13^C, Bruker, Fällanden, Switzerland) using TMS as an internal standard. HRESIMS were measured on a Thermo MAT95XP high resolution mass spectrometer (Thermo Fisher Scientific, Bremen, Germany), and ESIMS spectra on a Thermo DSQ EImass spectrometer (Thermo Fisher Scientific, Bremen, Germany). Single-crystal X-ray crystallographic analysis was performed on a Bruker D8 venture X-ray single crystal diffractomete (Bruker, Karlsruhe, Germany). Samples were analyzed on a Hitachi L-2000 HPLC system, coupled with a Hitachi L-2455 photodiode array detector, and using a C18 column (Kromasil 250 × 4.6 mm, 5 µm, Nouryon, Bohus, Sweden). The semi-preparative HPLC was conducted by a semi-preparative C18 column (Kromasil 250 × 10 mm, 5 µm). Silica gel (Qing Dao Hai Yang Chemical Group Co., Qingdao, China; 300−400 mesh) and Sephadex LH-20 (Amersham Biosciences, Uppsala, Sweden) were used for column chromatography (CC). Precoated silica gel plates (Yan Tai Zi Fu Chemical Group Co., Yantai, China; G60, F-254) were used for thin-layer chromatography.

### 2.2. Fungal Material

The fungal strain *Metarhizium* sp. P2100 was isolated from the seawater collected from the Qingdao Huiquan Bay, Yellow Sea, in August 2019. The fungus was identified as *Metarhizium* sp. fungus based on its morphological features and the sequence analysis of the internally transcribed spacer (ITS) region (GenBank accession number: OP028052) of the rRNA gene, as well as its derived phylogenetic analysis [24]. This fungal strain was deposited in the Key Laboratory of Marine Drugs, Ministry of Education of China, School of Medicine and Pharmacy, Ocean University of China, Qingdao, China.

### 2.3. Fungal Fermentation and Metabolite Profile Analysis

The fungal strain was cultured on a rice solid medium (100 × 1000 mL erlenmeyer flasks, each containing rice: 80 g, yeast extract: 0.8 g, peptone: 0.8 g, glucose: 1.6 g, sea salt: 4 g, and water: 80 mL.) for 30 days at 25 °C. The fermented rice substrate was extracted three times with ethyl acetate (EtOAc) and concentrated under vacuum evaporation to yield an organic extract (48.1 g). The EtOAc extract was eluted on silica gel column chromatography (CC) using a step gradient elution with petroleum ether/ethyl acetate (10:0 to 0:10, *v*/*v*) and ethyl acetate/methanol (10:0 to 0:10) to provide eight fractions (Fr.1−Fr.8). Fr.7 was separated by silica gel CC eluted with dichloromethane/methanol (80:1, *v*/*v*) and purified by the semi-preparative high performance liquid chromatography (HPLC), eluted with methanol/H_2_O (1:1, *v*/*v*) to give compound **1** (10.3 mg).

The fungal strain was cultured on rice-wheat solid medium (100 × 1000 mL erlenmeyer flasks, each containing wheat: 20 g, rice: 60 g, saccharose: 1.8 g, NaNO_3:_ 0.18 g, KH_2_PO_4_: 0.06 g, MgSO_4_.7H_2_O: 0.03 g, KCl: 0.03 g, FeSO_4_: 0.0006 g, sea salt: 1.2 g and water: 60 mL) for 30 days at 25 °C. The fermented rice substrate was extracted three times with EtOAc and concentrated under vacuum evaporation to yield an organic extract (43.5 g). The EtOAc extract was eluted on silica gel CC using a step gradient elution with petroleum ether/ethyl acetate (10:0 to 0:10, *v*/*v*) and ethyl acetate/methanol (10:0 to 0:10) to provide ten fractions (Fr.1−Fr.10). Fr.8 was separated by silica gel CC using a step gradient elution with petroleum ether/ethyl acetate (2:1, 1:1, 1:2 and 0:1, *v*/*v*) to provide six fractions (Fr.8.1−Fr.8.6). Fr.8.4 was separated by silica gel CC eluted with petroleum ether/ethyl acetate (1:5, *v*/*v*) to obtain compound **3** (73.0 mg). Fr.8.5 was separated by Sephadex LH-20 CC, eluted with dichloromethane/methanol (1:1, *v*/*v*) and the semi-preparative HPLC eluted with methanol/H_2_O (2:3, *v*/*v*), to yield compound **2** (20.3 mg) and **4** (6.7 mg).

### 2.4. ECD Calculation of Metabolites

Monte Carlo conformational searches were carried out via the Spartan’s software (Wavefunction&Q-Chem, Irvine&,Pleasanton USA) using the Merck Molecular Force Field (MMFF). The conformers with a Boltzmann population of over 5% were chosen for ECD calculations, and initially optimized at B3LYP/6-311+G (d) level. The theoretical calculation of ECD was conducted using the Time Dependent Density Functional Theory (TDDFT) at the B3LYP/6-311+ +G (2d, p) level for all conformers. ECD spectra were generated using the program SpecDis 1.6 (University of Würzburg, Würzburg, Germany), according to Boltzmann distributions.

### 2.5. Bioassay

The antibacterial activity was evaluated following the standards recommended by Yang [25]. Three marine-derived pathogenic bacterial strains, *Vibriovulnificus* MCCC E1758, *V. rotiferianus* MCCC E385 and *V. campbellii* MCCC E333, were used, and ampicillin sodium was tested as a positive control.

The antifungal bioassay was conducted following the standards recommended by Yang [25]. Three pathogenic fungal strains, *Candida albicans* ATCC 24433, *C. tropicalis* ATCC 20962, and *C. parapsilosis* ATCC 22019 were tested, and with amphotericin B as a positive control.

The 1,1-diphenyl-2-picryl-hydazyl (DPPH) scavenging assay was performed using the method described by Aquino [26]. The reaction mixture consisted of freshly prepared 100 µmol/L DPPH in ethanol and different concentrations of the tested compound. The reaction mixtures were incubated for 20 min at room temperature in the dark, and the absorbance was recorded at 517 nm.

The Fe^3+^ reduction assay was performed using the method described by Aktumsek [27]. The reaction mixture consisted of freshly prepared 100 µmol/L 2,4,6-tripyridin-2-yl-1,3,5-triazine (TPTZ) in ultrapure water and different concentrations of tested compounds. The reaction mixture was incubated for 20 min at room temperature in the dark, and the absorbance was recorded at 593 nm.

The bioassay for NO production inhibitory activity was conducted as described by Xia [28]. The mouse macrophage was seeded in 96-well plates. In each well, lipopolysaccharide (LPS) (1 μg/mL) was added after treating it with or without the tested compound for 24 h. The NO production in the supernatant was detected by the Griess reaction. The absorbance at 540 nm was measured with a microplate reader. The NO concentration and the inhibitory rate were calculated through a calibration curve. Dexamethasone was used as the positive control. Experiments were operated in triplicate, and the data were described as the mean ± SD of three independent experiments.

The *α*-amylase inhibition activity was measured by Milella [29] with minor modifications. Tested compounds and 1-Deoxynojirimycin (1-DNJ) were dissolved in methanol at different concentrations. A substrate solution was prepared with 0.25% starch dissolved in 20 mM phosphate buffer solution (PBS). The enzyme (10 units/mL of α-amylase in PBS, 25 µL) and sample solutions (25 µL) were mixed in Eppendorf vials, and the mixtures were incubated at 37 °C for 10 min. Then, 25 µL of substrate solution was added to each tube, and the reaction mixture was incubated at 37 °C for 10 min. The reaction was terminated by adding 150 µL of dinitrosalicylic acid color reagent. Subsequently, the test tubes were heated for 5 min at 100 °C. After cooling at room temperature, the absorbance was measured at 540 nm. The absorbance of the blank (enzyme solution was added during the boiling) and negative controls (methanol) were recorded. Analyses were performed in triplicate, and the final sample absorbance was obtained by subtracting its corresponding blank reading. The inhibitory activity (%) was calculated as follows:

$$\%\;\mathrm{inhibition}=(1-\frac{A_{540\;\mathrm{sample}}}{A_{540\;\mathrm{negative}\;\mathrm{control}}})\;\times \;100\%$$

The half-maximal inhibitory concentration (IC_50_) values were calculated according to the inhibition curve and the data were shown in the layout of mean ± SEM by GraphPad Prism 6.0 (GraphPad Software, Inc., La Jolla, CA, USA).

## 3. Results and Discussions

**Figure 1:**
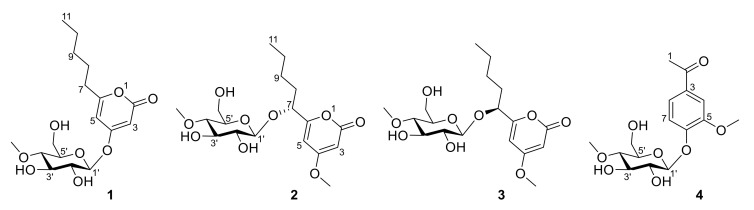
Structures of compounds **1**–**4**.

From the ethyl acetate extracts of the fermented rice solid and rice-wheat solid media of *Metarhizium* sp. P2100, four new glucoside compounds (**1**–**4**) (Figure 1) were isolated by column chromatography and HPLC. Compound **1** is a pyranone glucoside whose C4 position is substituted by a 4-*O*-methyl-*β*-D-glucose. Compounds **2** and **3** are a pair of epimeric pyranoside glycosides with a 4-*O*-methyl-*β*-D-glucose attached to their C7 positions. Compound **4** is a phenolic glucoside.

**Figure 2:**
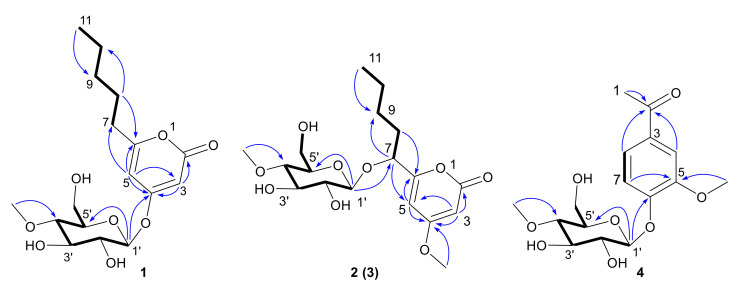
Key ^1^H-^1^H COSY (bold stripe) and HMBC (blue arrows) correlations of compounds **1**–**4**.

Compound **1** was obtained as a colorless crystalline. Its molecular formula (C_17_H_26_O_8_) was deduced from its HRESIMS at 381.1531 [M + Na]^+^ (calcd for 381.1520), indicating five degrees of unsaturation. Its IR spectrum displayed absorption bands for hydroxyl (3340 cm^−1^) and unsaturated lactone (1707 cm^−1^) groups. The ^1^H and ^13^C NMR spectra (see Table 1) of **1** were similar to the reported data for 4-methoxy-6-pentyl-2*H*-pyran-2-one [30]. The differences between **1** and 4-methoxy-6-pentyl-2*H*-pyran-2-one were the additional signals (*δ*_H_/*δ*_C_: 5.03/100.7 H-1′/C-1′; 3.44/74.5 H-2′/C-2′; 3.56/77.7 H-3′/C-3′; 3.19/80.1 H-4′/C-4′; 3.48/77.5 H-5′/C-5′; 3.83, 3.69/61.7 H-6a′, H-6b′/C-6′, and 3.48/60.9 4′-OMe) and the lack of signals in C4 of an methoxy, which were attributed to an additional methyl-hexose moiety. It was speculated that compound **1** should be a glycoside derivative of *α*-pyranone, with the sugar residue attached at C4. The ^1^H-^1^H COSY spectra revealed the presence of H-7/H-8, H-8/H-9, H-9/H-10, H-10/H-11 coupling, and the HMBC correlations from H-3 to C-2/C-4, H-5 to C-3/C-6/C-7 indicated the presence of 6-pentyl-2*H*-pyran-2-one unit. In addition, the ^1^H-^1^H COSY correlations of H-1′/H-2′, H-2′/H-3′, H-3′/H-4′, H-4′/H-5′, H-5′/H-6′ and the HMBC correlations from H-1′ to C-5′, 4′-OMe to C-4 revealed the existence of a 4-*O*-methyl-sugar residue. In addition, a strong HMBC interaction between H-1′ and C-4 confirmed that the sugar unit was attached to the hydroxy group at C4 through an *O*-glycosidic linkage. Thus, the planar structure of **1** was completely defined, as shown in Figure 2 (for spectrums of Compound 1 see Figures S1–S7).

**Figure 3:**
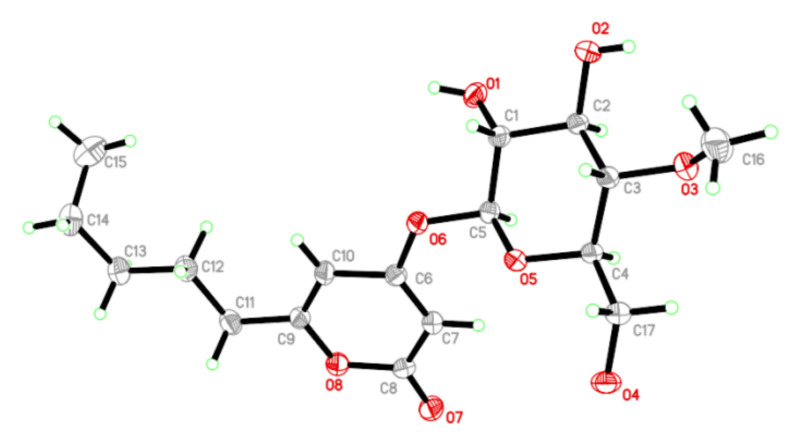
Single-crystal X-ray diffraction of **1**. Note: a different numbering system is used for the structural data (red circle: oxygen atom; blue circle: nitrogen atom).

The absolute configuration of compound **1** was confirmed by single-crystal X-ray diffraction using Cu *Kα* radiation. The perspective oak ridge thermal ellipsoid plot (ORTEP) plot is shown in Figure 3. Thus, the sugar residue was confirmed as 4-methoxy-*β*-D-glucosyl and allowed an unambiguous assignment of the absolute configuration as 1′*S*,2′*R*,3′*R*,4′*S*,5′*R*, therefore compound **1** was characterized as 4-(4′-methoxy-*β*-D-glucosyl)-6-pentyl-2-pyrone.

Compound **2** was obtained as a yellow oil. Its molecular formula (C_18_H_28_O_9_) was deduced from its HRESIMS at 389.1807 [M+H] ^+^ (calcd for 389.1767), indicating 5 degrees of unsaturation. Its IR spectrum displayed absorption bands for hydroxyl (3345 cm^−1^) and unsaturated lactone (1695 cm^−1^) groups. The ^1^H and ^13^C NMR spectra (see Table 1) suggested that **2** was very similar to compound **1**. The difference between **2** and **1** was the connecting position of 4-methoxy-*β*-D-glucosyl. The HMBC correlation from H-1′ to C-7 revealed that the sugar unit was attached to the hydroxy group at C7 through an *O*-glycosidic linkage. The HMBC correlation of methoxy group to C-4 revealed that the methoxy was attached at C4. The sugar was characterized as 4-*O*-methyl-*β*-D-glucose based on the coupling pattern observed for H-1′ (*δ*_H_ 4.36, d, *J* = 7.8 Hz), H-2′ (*δ*_H_ 3.22, dd, *J* = 9.3, 7.8 Hz), and H-3′ (*δ*_H_ 3.44, t, *J* = 9.0 Hz), which are consistent with compound **1**. Thus, the planar structure of **2** was completely defined (for spectrums of compound 2 see Figures S8–S15).

**Figure 4:**
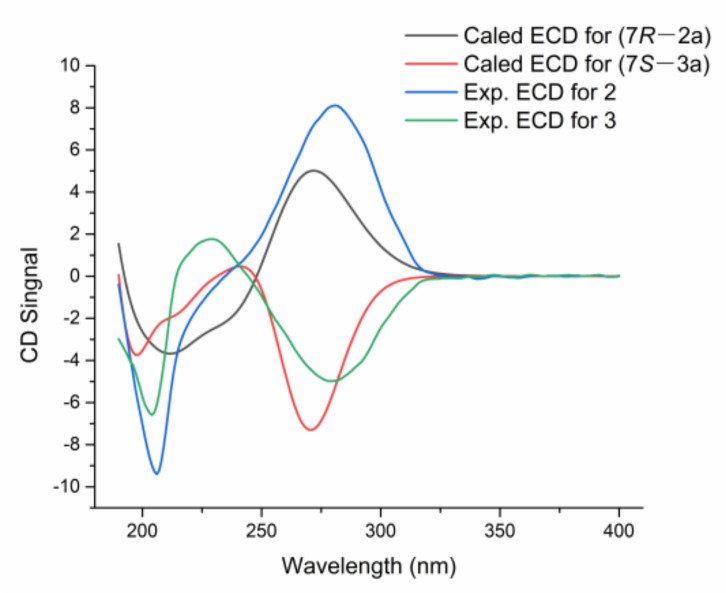
Experimental ECD spectra of **2**/**3** and calculated ECD spectra of **2a**/**3a**.

Compound **3** had the same molecular formula as **2** (C_18_H_28_O_9_), which was deduced from its HRESIMS with 389.1808 [M+H] ^+^ (calcd for 389.1767). Comparing the 1D- and 2D-NMR spectra of **3** with those of **2** (see Table 1) suggested that they might be a pair of epimers. The main differences were the ^1^H and ^13^C NMR signals of positions at C-7, C-5, and C-1′. Notably, the Cotton effect at 280 nm in the ECD spectrum of **3** was opposite to that of **2** (Figure 4). Compound **3** was presumed to adopt the opposite configuration to compound **2** at C7. Thus, the planar structure of **3** was completely defined (For spectrums of compound 3 see Figures S16–S23).

**Figure 5:**
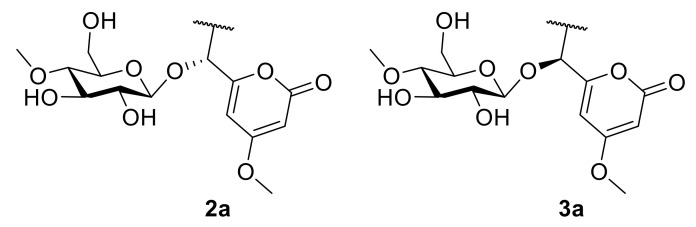
The structures of truncated model compounds **2a** and **3a**.

The absolute configurations of **2** and **3** were determined by ECD calculation. Due to the present of an alkyl side chain in compounds **2** and **3**, their structures possess high conformational flexibility and low convergence, so it is difficult to search and calculate for all their conformations. In fact, the alkyl side chain has a negligible impact on the ECD spectrum, since there is no chirality in the alkyl side chain. Thus, the simplified truncated model compounds **2a** and **3a** (Figure 5) were adopted by removing the alkyl side chains of **2** and **3**, which were used for conformational analysis and ECD calculations. Truncated model compounds of the original structures are often used in TDDFT-ECD calculations to simplify the conformational analysis without changing the computed Boltzmann-weighted ECD spectrum [31,32]. The calculations for the **2a** and **3a** electronic circular dichroism (ECD) spectra were performed by using the time-dependent density functional theory (TDDFT) at the B3LYP/6-311+G (d, p) level in methanol. The results showed that the calculated ECD spectra of 7*R* and 7*S* for **2a** and **3a** had positive and negative Cotton effects in the range of 250−330 nm, respectively, which were in accordance with the experimental spectra of **2** and **3** (Figure 4). Thus, the absolute configurations of compounds **2** and **3** were defined as 7*R* and 7*S*, respectively. Thus, compounds **2** and **3** were determined as a pair of epimeric pyranoside glycosides, and named as 7(*R*)-(4′-methoxy-*β*-D-glucosyl)-4-methoxy-6-pentyl-2-pyrone (**2**) and 7(*S*)-(4′-methoxy-*β*-D-glucosyl)-4-methoxy-6-pentyl-2-pyrone (**3**), respectively.

Lecaniside D (**4**) was obtained as a yellow oil. Its molecular formula (C_16_H_22_O_8_) was deduced from its HRESIMS at 365.1199 [M+Na] ^+^ (calcd for 365.1207), indicating 6 degrees of unsaturation. The IR spectrum displayed absorption bands for hydroxyl (3319 cm^−1^), phenyl ring and ketone (1668, 1593cm^−1^) groups. The ^1^H and ^13^C NMR spectra (Table 2) were very similar to the reported data of lecaniside C, isolated from *Lecanicillium attenuatum* [33]. Compared to lecaniside C, the ^13^C NMR spectrum of compound **4** disappeared a carbon signal at *δ*_C_ 8.4. The HMBC correlations from H-1 to C-2, H-4 to C-2, and H-8 to C-2 indicated that one acetyl group should be attached to C-3 in compound **4**, instead of a propionyl group in lecaniside C. Likewise, one sugar anomeric carbon at *δ*_C_ 101.66 (Glc1′-C) showed a sugar substituent. Meanwhile, this sugar was characterized as 4-*O*-methyl-*β*-D-glucose on the basis of the coupling pattern observed for H-1′ (*δ*_H_ 5.02, d, *J* = 7.3 Hz) and H-4′ (*δ*_H_ 3.21, dd, *J* = 9.8, 8.4 Hz). Based on the similarity between compound **4** and lecaniside C, compound **4** was named lecaniside D (For spectrums of Compound 4 see Figures S24–S31).

These isolated glucoside compounds were evaluated for their antibacterial, antifungal, DPPH scavenging activities, Fe^3+^ reduction, cell proliferation inhibition, *α*-amylase inhibition, and anti-inflammatory activities. No compounds showed antibacterial, antifungal, DPPH scavenging activity and Fe^3+^ reduction ability. Compounds **1**–**4** exhibited weak anti-inflammatory activities, and compounds **2**–**3** showed weak cell proliferation inhibition activities (Table 3). Compounds **1**–**3** exhibited inhibitory activities against *α*-amylase with the IC_50_ values of 128, 64 and 64 μM, respectively, slightly weaker than acarbose with the IC_50_ values of 26.3 ± 1.2 μM [29]. Several anologs of **1**–**4** had been reported previously. 4′-O-methyl-β-mannopyranoside, isolated from *Xylaria feejeensis*, showed α-glucosidase inhibitory activity [34]. Lecaniside C, an analog of **4**, has PTP1B inhibitory activity with the IC50 > 50 μM [29].

4-(4′-methoxy-*β*-D-glucosyl)-6-pentyl-2-pyrone (**1**): colorless crystalline; mp 162–167 °C; [α]^25^_D_ –55.8 (*c* 1.0, MeOH); UV (MeOH) λ_max_ (log *ε*) 208 (3.94), 287 (3.77) nm; CD (MeOH) λ_max_ (Δε) 196 (–7.15), 245 (–0.09), 287 (–1.33); IR (KBr)*ν*_max_ 2930, 1707, 1246, 1081 cm^–1^; ^1^H (400 MHz, methanol-*d*_4_) and ^13^C NMR (100 MHz, methanol-*d*_4_) see Table 1; HREMIMS *m*/*z* 381.1531 [M+Na]^+^ (C_17_H_26_O_8_Na, calcd for 381.1520).

Crystal date for compound **1**: C_17_H_26_O_8_, *Mr* = 358.38, Identification code: 12257, Temperature: 293(2) K, Crystal system: monoclinic, Space group: P21, *a* =4.9520(3) Å, *b* = 13.0099(6) Å, *c* = 13.8611(7)Å, *α* = 90°, *β* = 92.644(5)°, *γ* = 90°, Volume 892.05(8) Å^3^, Z = 2, *ρ*_calcg_/cm^3^ 1.334, *μ* = 0.892 mm^–1^, F(000) = 384.0, Crystal size: 0.120 × 0.120 × 0.110 mm^3^, Radiation Cu *Kα* (*λ* = 1.54178), Reflections collected: 3044, Independent reflections: 2278 (*R*_int_ = 0.0312, *R*_sigma_ = 0.0486), Data/restraints/parameters: 2278/1/231, Goodness-of-fit on F^2^ = 1.079, Final *R* indexes (*I* >= 2*σ* (*I*)) *R*_1_ = 0.0470, *wR*_2_ = 0.1194, Final *R* indexes (all data) *R*_1_ = 0.0528, *wR*_2_ = 0.1261, Largest different peak/hole = 0.13/–0.21 e Å^–3^, Flack parameter = 0.0(3).

7(*R*)-(4′-methoxy-*β*-D-glucosyl)-4-methoxy-6-pentyl-2-pyrone (**2**): yellow oil; [α]^25^_D_ 57.3 (*c* 6.0, MeOH); UV (MeOH) λ_max_ (log *ε*) 206 (3.72), 280 (3.24) nm; CD (MeOH) λ_max_ (Δε) 206 (–9.45), 280 (8.17); IR (KBr)*ν*_max_ 3345, 2954, 1696, 1567, 1457, 1250, 1082 cm^–1^; ^1^H (400 MHz, methanol-*d*_4_) and ^13^C NMR (100 MHz, methanol-*d*_4_) see Table 1; HREMIMS *m*/*z* 389.1807 [M+H]^+^ (C_18_H_29_O_9_, calcd for 389.1767).

7(*S*)-(4′-methoxy-*β*-D-glucosyl)-4-methoxy-6-pentyl-2-pyrone (**3**): yellow oil; [α]^25^_D_ –79.9 (*c* 2.3, MeOH); UV (MeOH) λ_max_ (log *ε*) 203 (3.94), 280 (3.71) nm; CD (MeOH) λ_max_ (Δε) 203 (–6.75), 228 (1.78), 280 (–4.50); IR (KBr)*ν*_max_ 3393, 2955, 1700, 1568, 1457, 1249, 1083 cm^–1^; ^1^H (400 MHz, methanol-*d*_4_) and ^13^C NMR (100 MHz, methanol-*d*_4_) see Table 1; HREMIMS *m*/*z* 389.1808 [M+H]^+^ (C_18_H_29_O_9_, calcd for 389.1767).

Lecaniside D (**4**): yellow oil; [α]^25^_D_ –213. (*c* 2.5, MeOH); UV (MeOH) λ_max_ (log *ε*) 228 (4.17), 274 (3.91), 301 (3.74) nm; CD (MeOH) λ_max_ (Δε) 195 (–1.60), 208 (0.62), 225 (–4.20), 247 (–6.94), 272 (0.51), 300 (–1.75); IR (KBr)*ν*_max_ 3319, 2942, 2359, 2337, 1668, 1593, 1272, 1026 cm^–1^; ^1^H (400 MHz, methanol-*d*_4_) and ^13^C NMR (100 MHz, methanol-*d*_4_) see Table 2; HREMIMS *m*/*z* 365.1199 [M+Na]^+^ (C_16_H_22_O_8_Na, calcd for 365.1207).

## 4. Conclusions

In summary, four new glycosides were obtained and identified from a marine *Metarhizium* species for the first time, including three pyranoside glycosides (**1**–**3**) and a phenolic glycoside (**4**). In 4-(4′-methoxy-*β*-D-glucosyl)-6-pentyl-2-pyrone (**1**), its C4 position is oxymethyl glucose. Meanwhile, 7(*R*)-(4′-methoxy-*β*-D-glucosyl)-4-methoxy-6-pentyl-2-pyrone (**2**) and 7(*S*)-(4′-methoxy-*β*-D-glucosyl)-4-methoxy-6-pentyl-2-pyrone (**3**) are a pair of rare epimeric pyranoside glycosides with the C-7 position attaching an oxymethyl glucose. Lecaniside D (**4**) is a phenolic glycoside with oxymethyl glucose. Compounds **1**–**4** exhibited weak anti-inflammatory activities, and compounds **1**–**3** showed inhibition activities against *α*-amylase. These data suggested that marine fungi should be a new source of *α*-amylase inhibitors and have potential in the treatment of diabetes.

## Figures and Tables

**Table 1:** ^1^H (400 MHz) and ^13^C (100 MHz) NMR data of compounds **1–3** in Methanol-*d*_4_.

No.	1		2		3	
	*δ*_H_ (*J* in Hz)	*δ*_C_	*δ*_H_ (*J* in Hz)	*δ*_C_	*δ*_H_ (*J* in Hz)	*δ*_C_
2		167.5		167.1		167.0
3	5.68 (d, *J* = 2.2 Hz, 1H)	91.7	5.57 (d, *J* = 2.3 Hz, 1H)	89.0	5.57 (d, *J* = 2.3 Hz, 1H)	89.1
4		171.4		173.7		173.5
5	6.11 (d, *J* = 2.2 Hz, 1H)	101.1	6.30 (d, *J* = 2.2 Hz, 1H)	101.8	6.51 (d, *J* = 2.2 Hz, 1H)	101.8
6		168.2		165.9		165.5
7	2.52 (t, *J* = 7.5 Hz, 2H)	34.4	4.44 (t, *J* = 6.3 Hz, 1H)	79.1	4.62 (dd, *J* = 7.2, 5.2 Hz, 1H)	76.4
8	1.66 (p, *J* = 7.4 Hz, 2H)	27.6	1.84 (td, *J* = 6.3, 2.2 Hz, 2H)	33.5	1.89–1.70 (m, 2H)	34.7
9	1.34 (m, 2H)	32.1	1.42–1.37 (m,2H)	27.8	1.42–1.37 (m, 2H)	28.0
10	1.36 (m, 2H)	23.4	1.37–1.27 (m, 2H)	23.6	1.37–1.26 (m, 2H)	23.4
11	0.92 (t, *J* = 6.9 Hz, 3H)	14.3	0.89 (m, 3H)	14.3	0.90 (t, *J* = 7.1 Hz, 3H)	14.3
4-OMe			3.86 (s, 3H)	57.0	3.87 (s, 3H)	57.0
1′	5.03 (d, *J* = 7.8 Hz, 1H)	100.7	4.36 (d, *J* = 7.8 Hz, 1H)	104.2	4.24 (d, *J* = 7.8 Hz, 1H)	102.1
2′	3.44 (dd, *J* = 9.4, 7.8 Hz)	74.5	3.22 (dd, *J* = 9.3, 7.8 Hz, 1H)	75.2	3.25 (dd, *J* = 9.2, 7.8 Hz, 1H)	75.1
3′	3.56 (dd, *J* = 9.4, 8.9 Hz, 1H)	77.7	3.44 (dd, *J* = 9.3, 8.8 Hz, 1H)	78.1	3.43 (dd, *J* = 9.2, 9.0 Hz, 1H)	78.0
4′	3.19 (dd, *J* = 9.8, 8.9 Hz, 1H)	80.1	3.08 (dd, *J* = 9.8, 8.8 Hz, 1H)	80.7	3.08 (dd, *J* = 9.6, 9.0 Hz, 1H)	80.9
5′	3.48 (m, 1H)	77.5	3.17 (m, 1H)	77.0	3.21 (m, 1H),	77.1
6′	3.83 (dd, *J* = 12.2, 2.2 Hz, 1H); 3.69 (dd, *J* = 12.2, 4.6 Hz, 1H)	61.7	3.68 (dd, *J* = 11.9, 2.1 Hz, 1H); 3.59 (dd, *J* = 11.9, 4.5 Hz, 1H)	62.2	3.67 (dd, *J* = 11.9, 5.2 Hz, 1H); 3.83 (dd, *J* = 11.9, 2.1 Hz, 1H)	62.3
4′-OMe	3.58 (s, 3H)	60.9	3.54 (s, 3H)	60.8	3.54 (s, 3H)	60.8

**Table 2:** The ^1^H (400 MHz) and ^13^C (100 MHz) NMR data of compound **4** in Methanol-*d*_4_.

No.	4	
	*δ*_H_ (*J* in Hz)	*δ*_C_
1	2.57 (s, 3H)	26.4
2		199.4
3		132.9
4	7.57 (d, *J* = 2.0 Hz, 1H)	112.4
5		150.6
6		152.4
7	7.20 (d, *J* = 8.5 Hz, 1H)	116.1
8	7.63 (dd, *J* = 8.5, 2.0 Hz, 1H)	124.4
5-OMe	3.90 (s, 3H)	56.6
1′	5.02 (d, *J* = 7.5 Hz, 1H)	101.7
2′	3.56 (dd, *J* = 9.4, 7.5 Hz, 1H)	74.8
3′	3.61 (dd, *J* = 9.4, 9.1 Hz, 1H)	77.9
4′	3.21 (dd, *J* = 9.2, 9.1 Hz, 1H)	80.5
5′	3.46 (m, 1H)	77.4
6′	3.83 (dd, *J* = 12.1, 2.6 Hz, 1H) 3.69 (dd, *J* = 12.1, 4.8 Hz 1H)	62.0
4′-OMe	3.59 (s, 3H)	60.9

**Table 3:** The results of anti-inflammatory activities with concentration of 50 μM.

No.	1	2	3	4	Dexamethasone
NO production inhibition ratio (%)	53.85 ± 2.32	62.30 ± 1.46	68.44 ± 3.75	68.10 ± 2.07	63.88 ± 2.07
Cell proliferation inhibition ratio (%)	97.8 ± 10.25	117.23 ± 24.88	84.81 ± 4.9	77.45 ± 2.95	94.17 ± 4.82

Note: Dexamethasone as positive control.

## Data Availability

Not applicable.

## Supplementary Materials

The following supporting information can be downloaded at: https://www.mdpi.com/article/10.3390/jof9010028/s1. Figures S1–S7: ^1^H NMR, ^13^C NMR, HSQC, ^1^H-^1^H COSY, HMBC, HRESIMS and IR spectra of compounds **1;** Figures S8–S15: ^1^H NMR, ^13^C NMR, HSQC, ^1^H-^1^H COSY, HMBC, NOESY, HRESIMS and IR spectra of compounds **2**; Figures S16–S23: ^1^H NMR, ^13^C NMR, HSQC, ^1^H-^1^H COSY, HMBC, NOESY, HRESIMS and IR spectra of compounds **3**; Figures S24–S31: ^1^H NMR, ^13^C NMR, HSQC, ^1^H-^1^H COSY, HMBC, NOESY, HRESIMS and IR spectra of compounds **4.**
