# Supplementary material for: Bioactive Alpha-Pyrone and Phenolic Glucosides from the Marine-Derived Metarhizium sp. P2100
Source: J Fungi (Basel). 2022 Dec 23;9(1):28. doi: 10.3390/jof9010028 (PMC9863027; doi:10.3390/jof9010028)
Supplement: Supplementary file 1 [file jof-09-00028-s001.zip › jof-2076172-supplementary.pdf]

## List of Supplementary Material

**Figure S1.** $^{1}$H NMR (400 MHz, Methanol-$d_4$) spectrum of compound **1**
**Figure S2.** $^{13}$C NMR (100MHz, Methanol-$d_4$) spectrum of compound **1**
**Figure S3.** The HSQC (Methanol-$d_4$) spectrum of compound **1**
**Figure S4.** The $^{1}$H-$^{1}$H COSY (Methanol-$d_4$) spectrum of compound **1**
**Figure S5.** The HMBC (Methanol-$d_4$) spectrum of compound **1**
**Figure S6.** The HRESIMS spectrum of compound **1**
**Figure S7.** The IR spectrum of compound **1**
**Figure S8.** $^{1}$H NMR (400 MHz, Methanol-$d_4$) spectrum of compound **2**
**Figure S9.** $^{13}$C NMR (100MHz, Methanol-$d_4$) spectrum of compound **2**
**Figure S10.** The HSQC (Methanol-$d_4$) spectrum of compound **2**
**Figure S11.** The $^{1}$H-$^{1}$H COSY (Methanol-$d_4$) spectrum of compound **2**
**Figure S12.** The HMBC (Methanol-$d_4$) spectrum of compound **2**
**Figure S13.** The NOESY (Methanol-$d_4$) spectrum of compound **2**
**Figure S14.** The HRESIMS spectrum of compound **2**
**Figure S15.** The IR spectrum of compound **2**
**Figure S16.** $^{1}$H NMR (400 MHz, Methanol-$d_4$) spectrum of compound **3**
**Figure S17.** $^{13}$C NMR (100MHz, Methanol-$d_4$) spectrum of compound **3**
**Figure S18.** The HSQC (Methanol-$d_4$) spectrum of compound **3**
**Figure S19.** The $^{1}$H-$^{1}$H COSY (Methanol-$d_4$) spectrum of compound **3**
**Figure S20.** The HMBC (Methanol-$d_4$) spectrum of compound **3**
**Figure S21.** The NOESY (Methanol-$d_4$) spectrum of compound **3**
**Figure S22.** The HRESIMS spectrum of compound **3**
**Figure S23.** The IR spectrum of compound **3**
**Figure S24.** $^{1}$H NMR (400 MHz, Methanol-$d_4$) spectrum of compound **4**
**Figure S25.** $^{13}$C NMR (100MHz, Methanol-$d_4$) spectrum of compound **4**
**Figure S26.** The HSQC (Methanol-$d_4$) spectrum of compound **4**
**Figure S27.** The $^{1}$H-$^{1}$H COSY (Methanol-$d_4$) spectrum of compound **4**
**Figure S28.** The HMBC (Methanol-$d_4$) spectrum of compound **4**
**Figure S29.** The NOESY (Methanol-$d_4$) spectrum of compound **4**
**Figure S30.** The HRESIMS spectrum of compound **4**
**Figure S31.** The IR spectrum of compound **4**

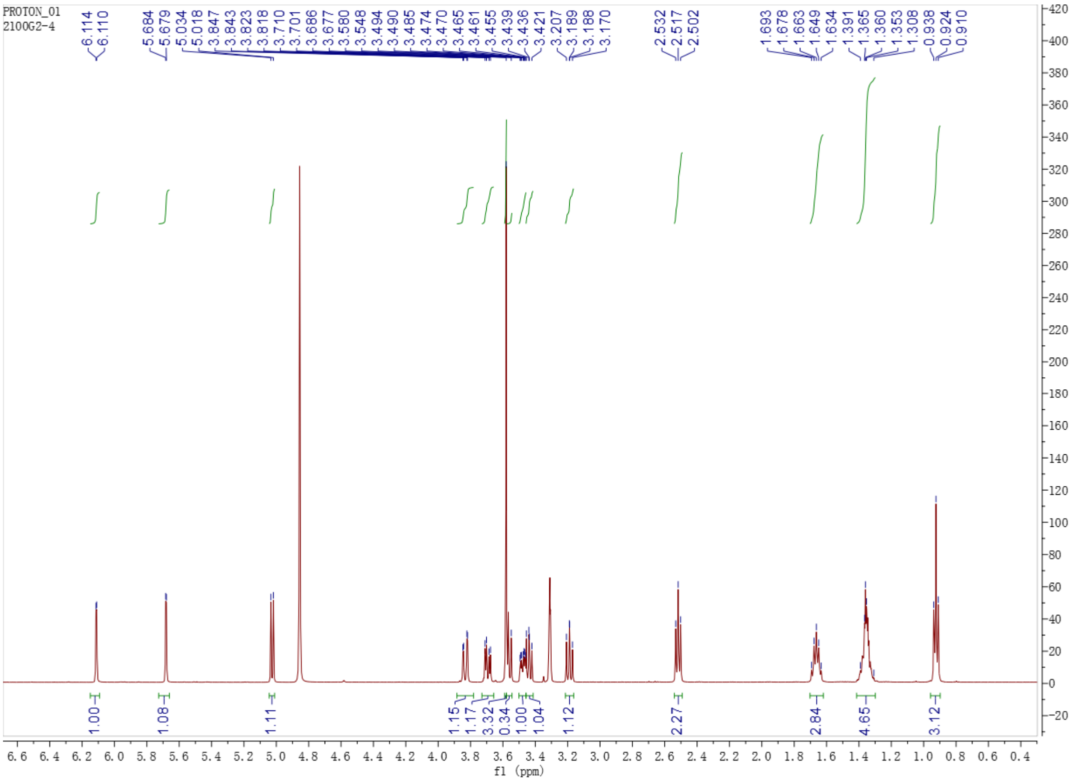


**Figure S1.** $^{1}H$ NMR (400 MHz, Methanol-$d_4$) spectrum of compound **1**

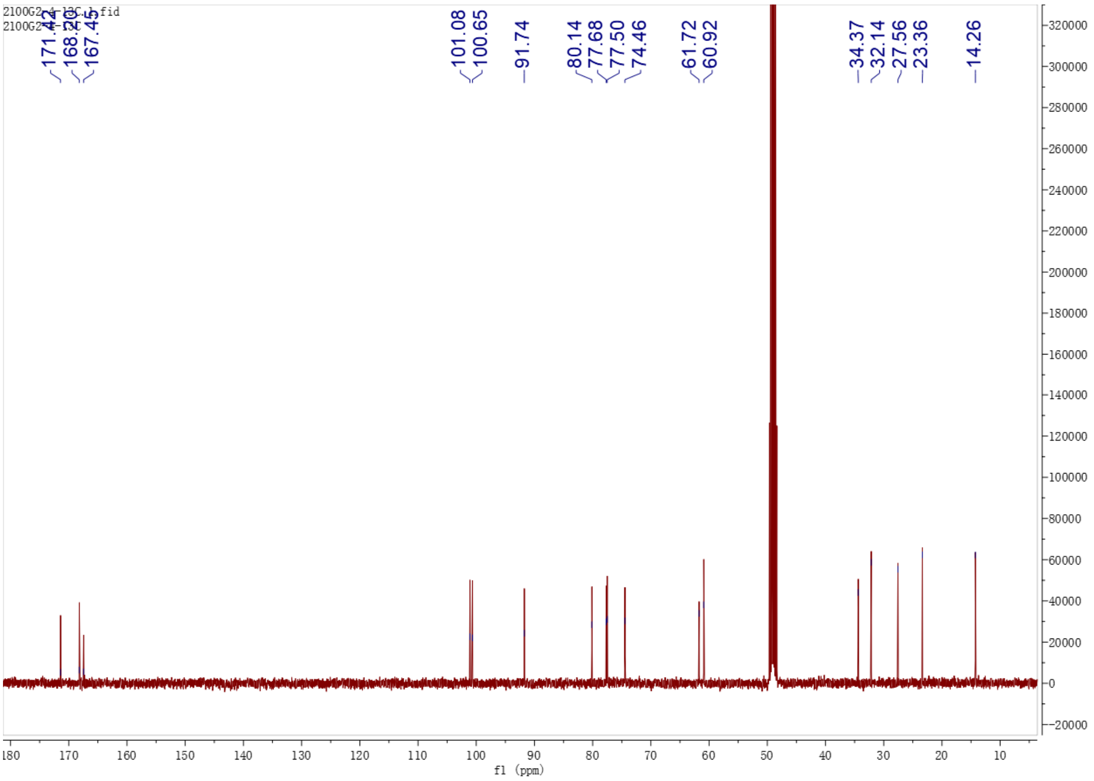


**Figure S2.** $^{13}C$ NMR (100MHz, Methanol-$d_4$) spectrum of compound **1**

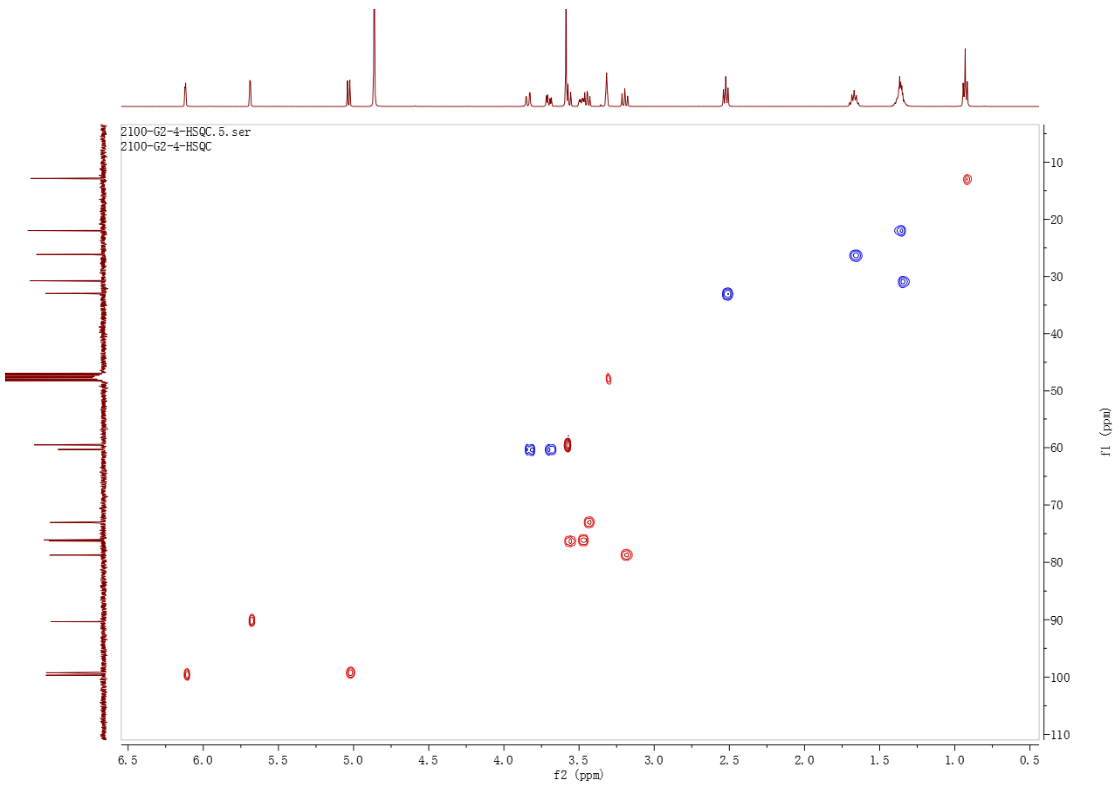


**Figure S3.** The HSQC (Methanol-$d_4$) spectrum of compound **1**

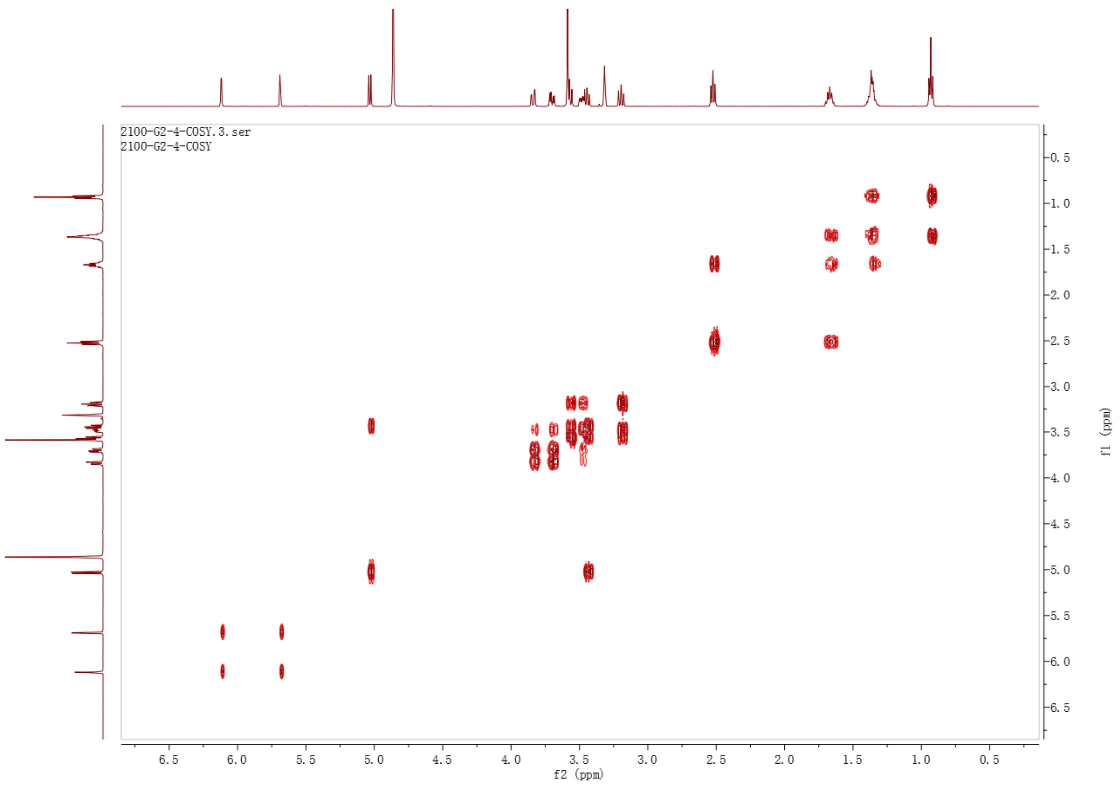


**Figure S4.** The $^1$H-$^1$H COSY (Methanol-$d_4$) spectrum of compound **1**

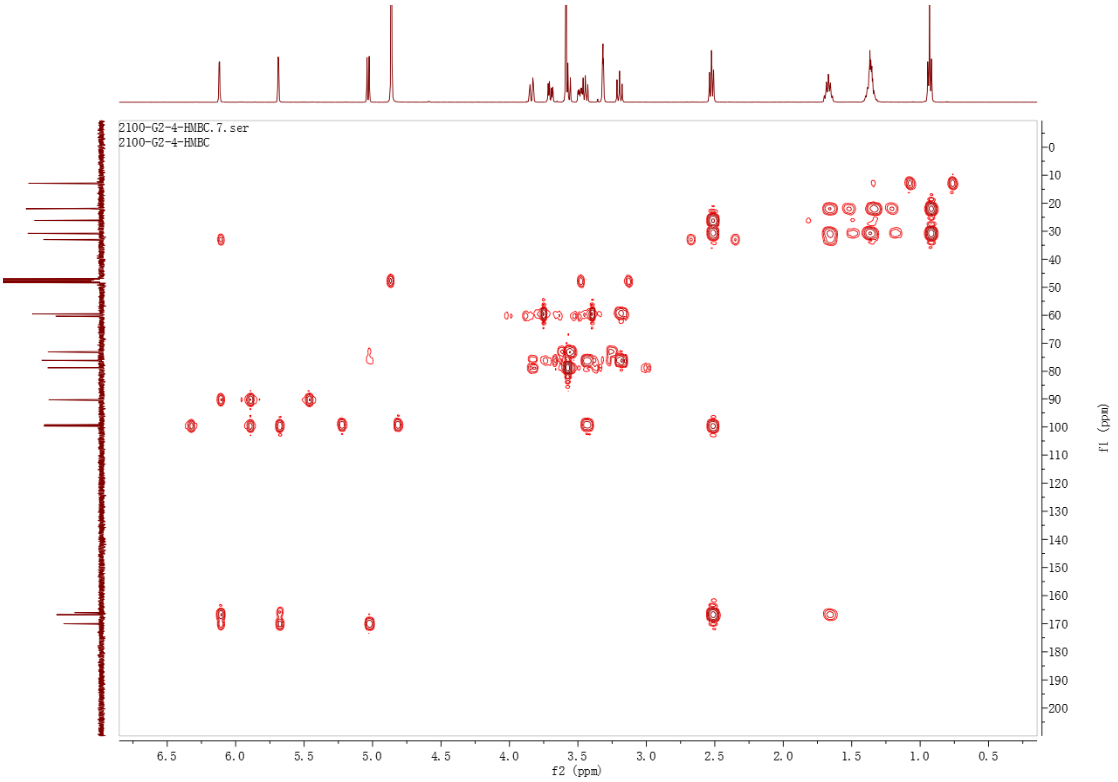


**Figure S5.** The HMBC (Methanol-*d*4) spectrum of compound **1**

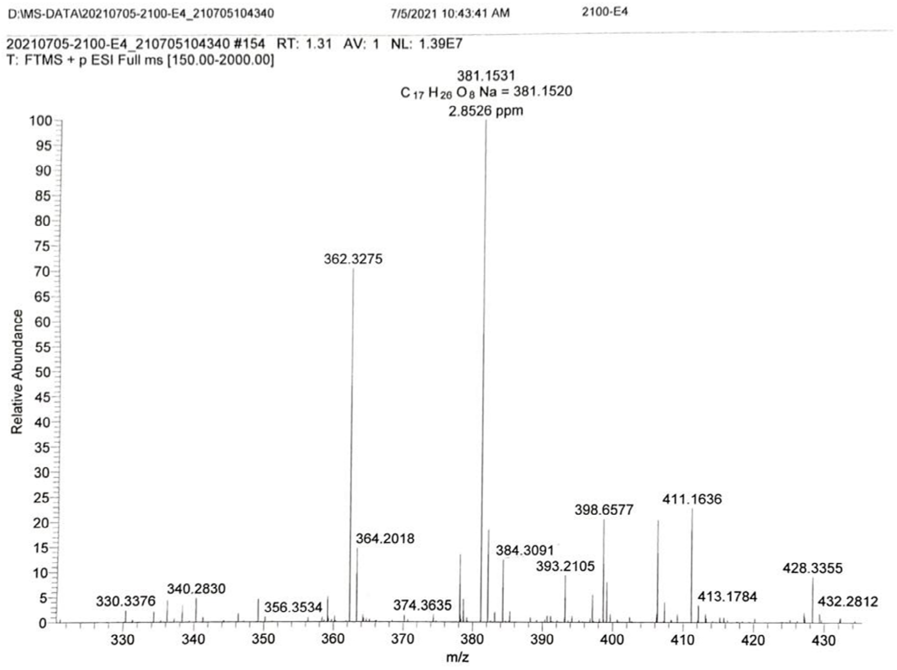


**Figure S6.** The HRESIMS spectrum of compound **1**

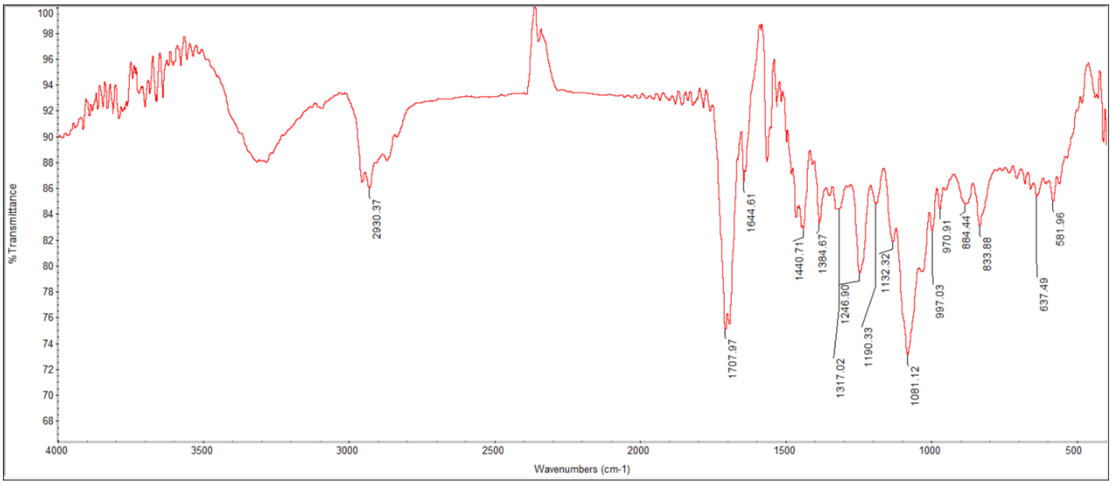

**Figure S7.** The IR spectrum of compound **1**

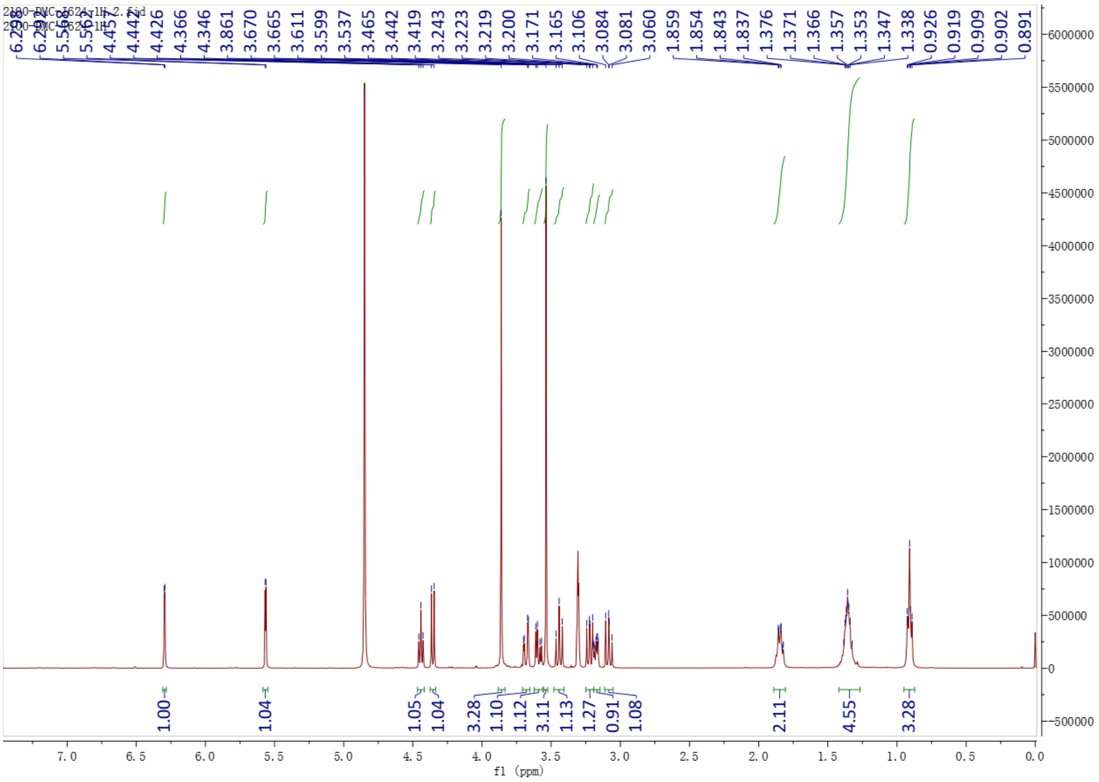

**Figure S8.** $^{1}$H NMR (400 MHz, Methanol-$d_4$) spectrum of compound **2**

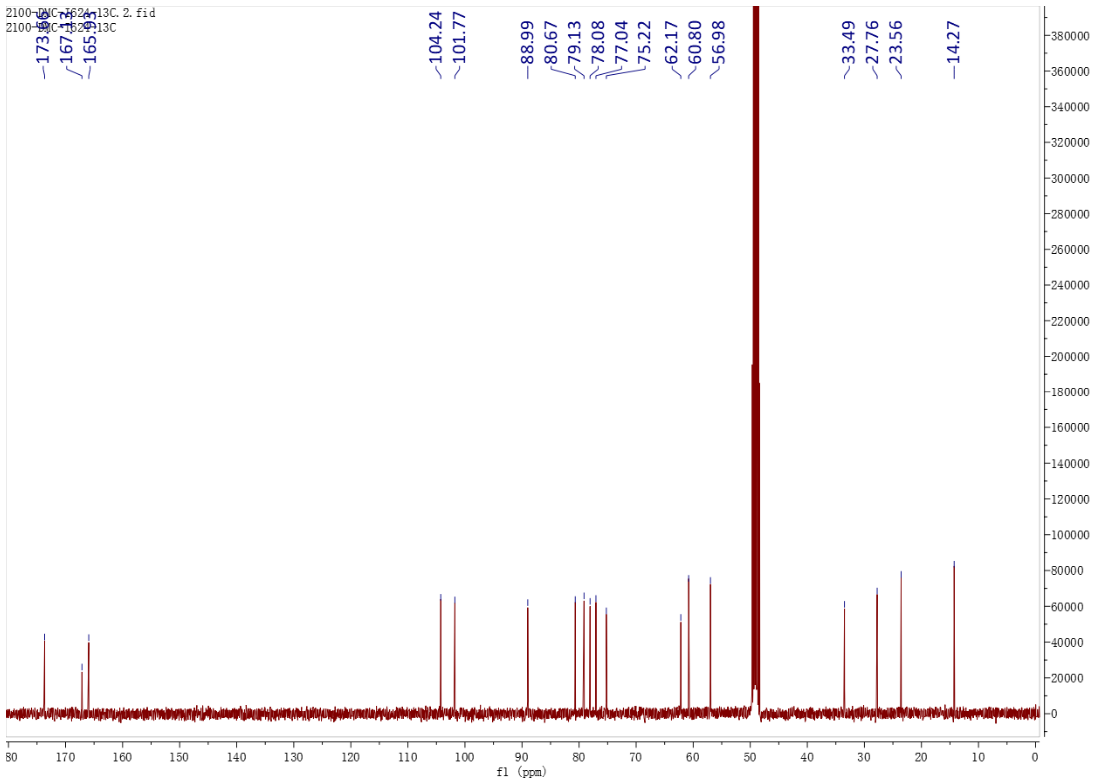


**Figure S9.** $^{13}C$ NMR (100 MHz, Methanol-*d*$_4$) spectrum of compound **2**

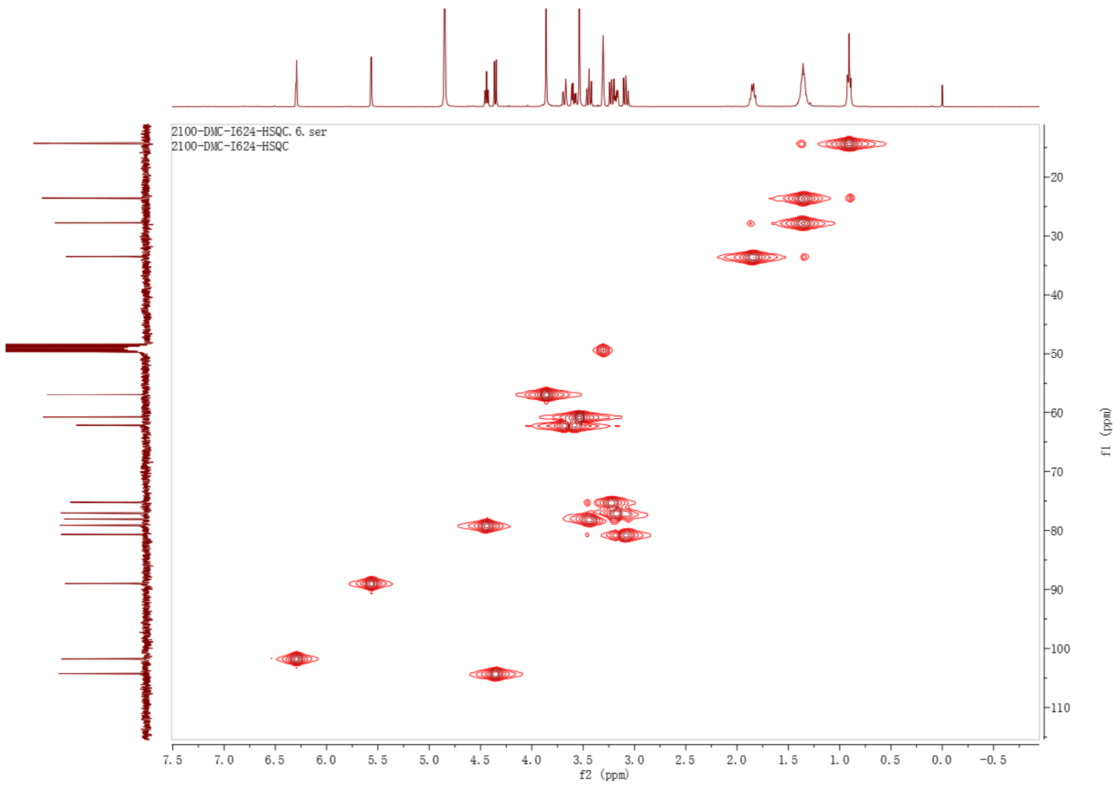


**Figure S10.** The HSQC ( Methanol-*d*$_4$) spectrum of compound **2**

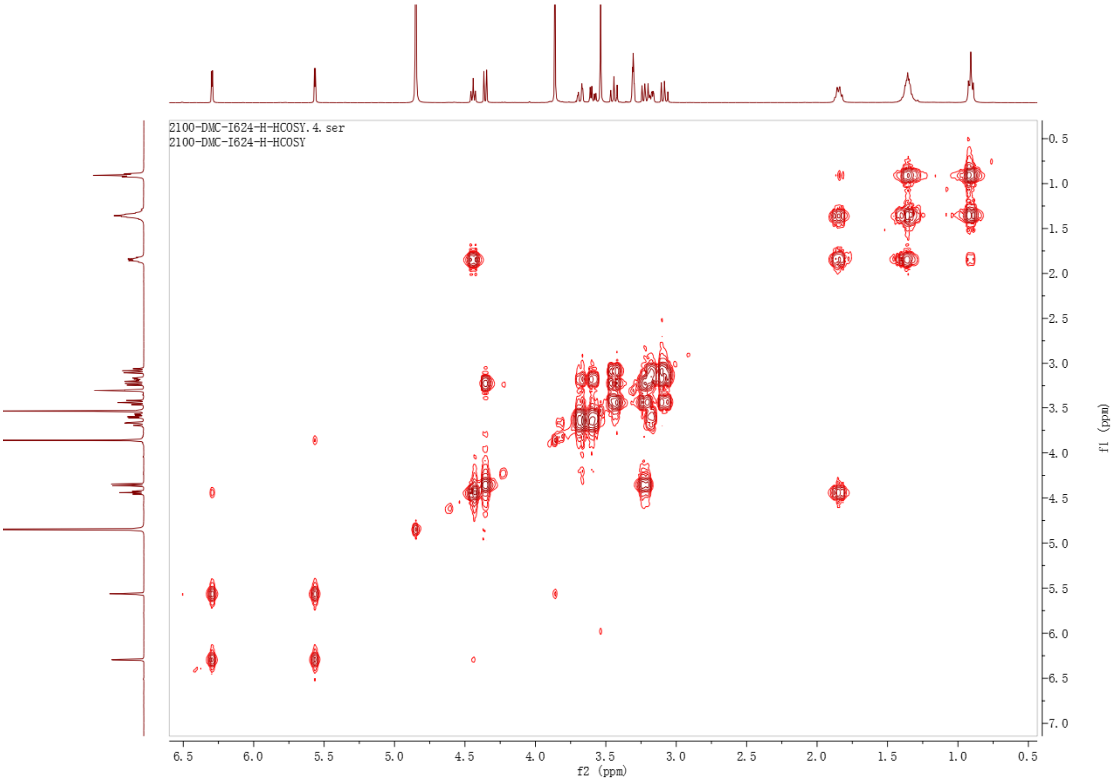


**Figure S11.** The $^{1}$H-$^{1}$H COSY (Methanol-*d*4) spectrum of compound **2**

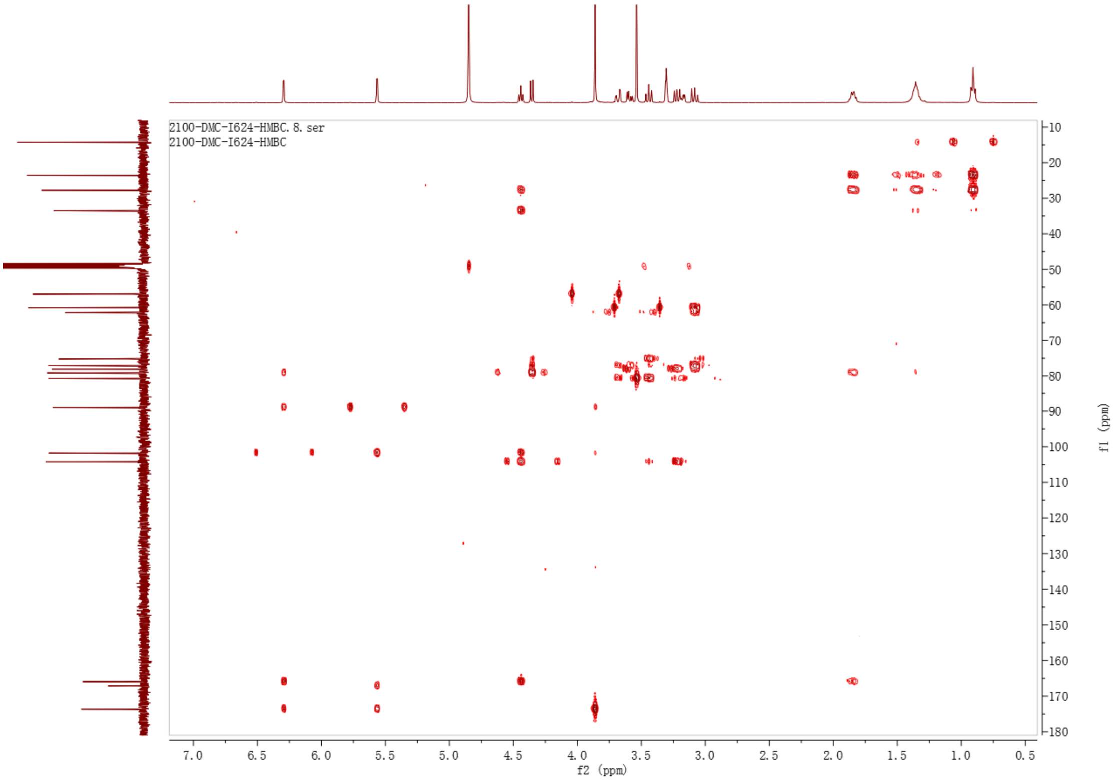


**Figure S12.** The HMBC (Methanol-*d*4) spectrum of compound **2**

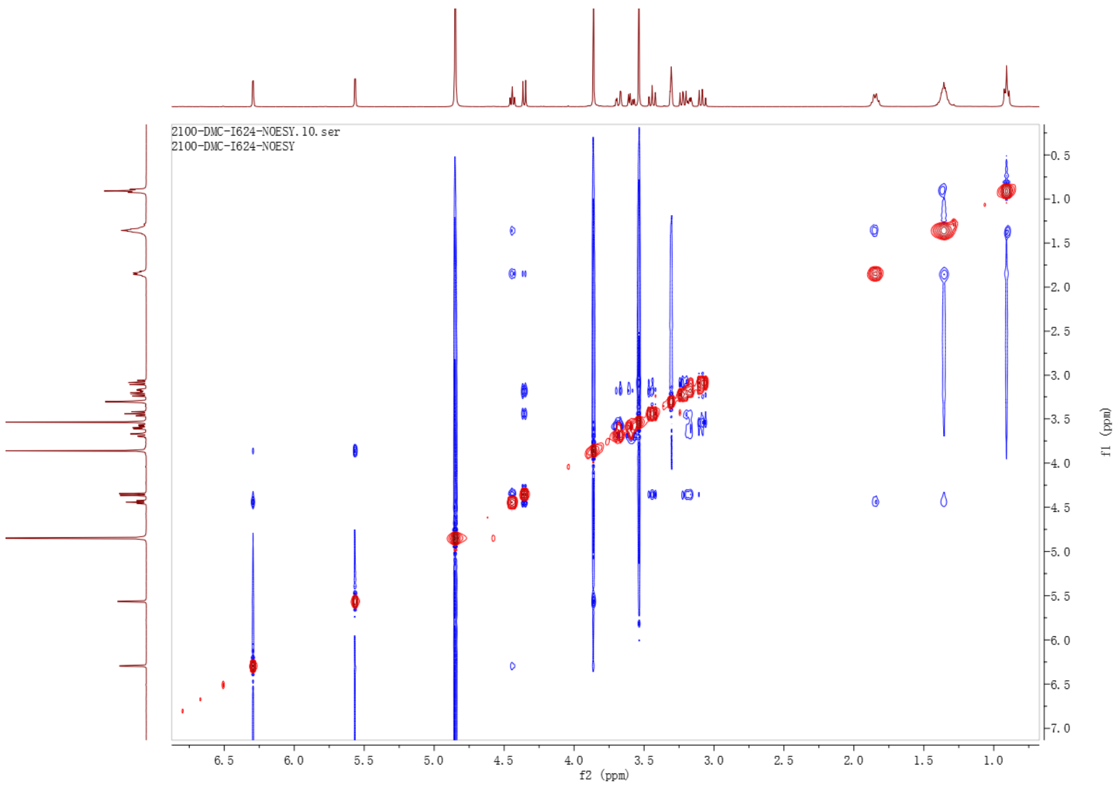


**Figure S13.** The NOESY (Methanol-$d_4$) spectrum of compound **2**

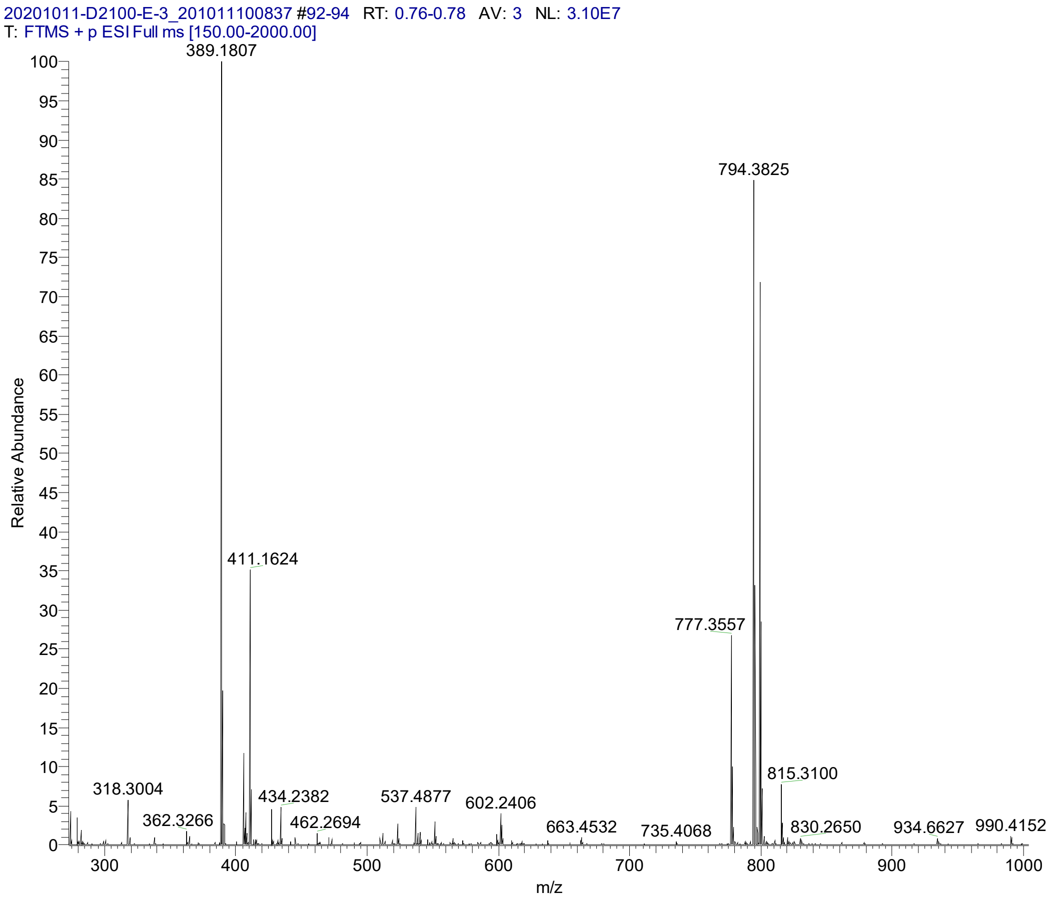


**Figure S14.** The HRESIMS spectrum of compound **2**

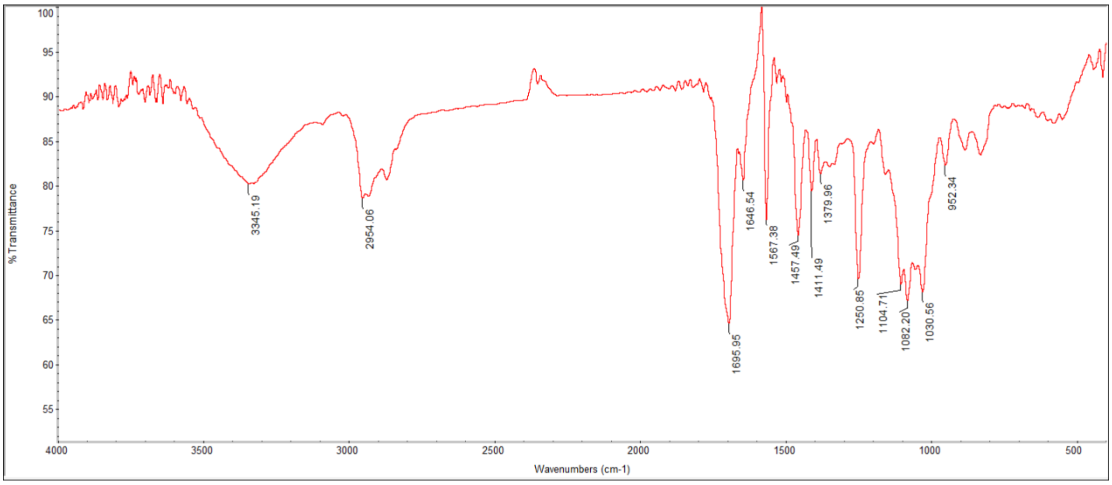


**Figure S15.** The IR spectrum of compound **2**

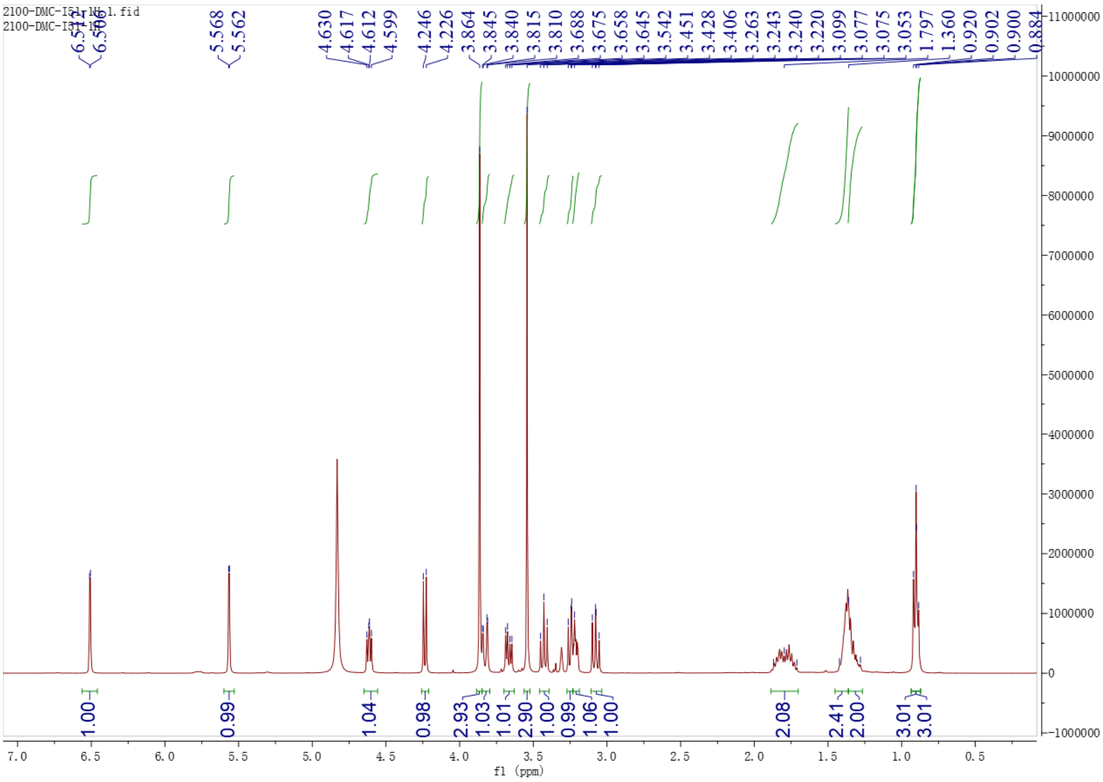


**Figure S16.** $^{1}$H NMR (400 MHz, Methanol-$d_4$) spectrum of compound **3**

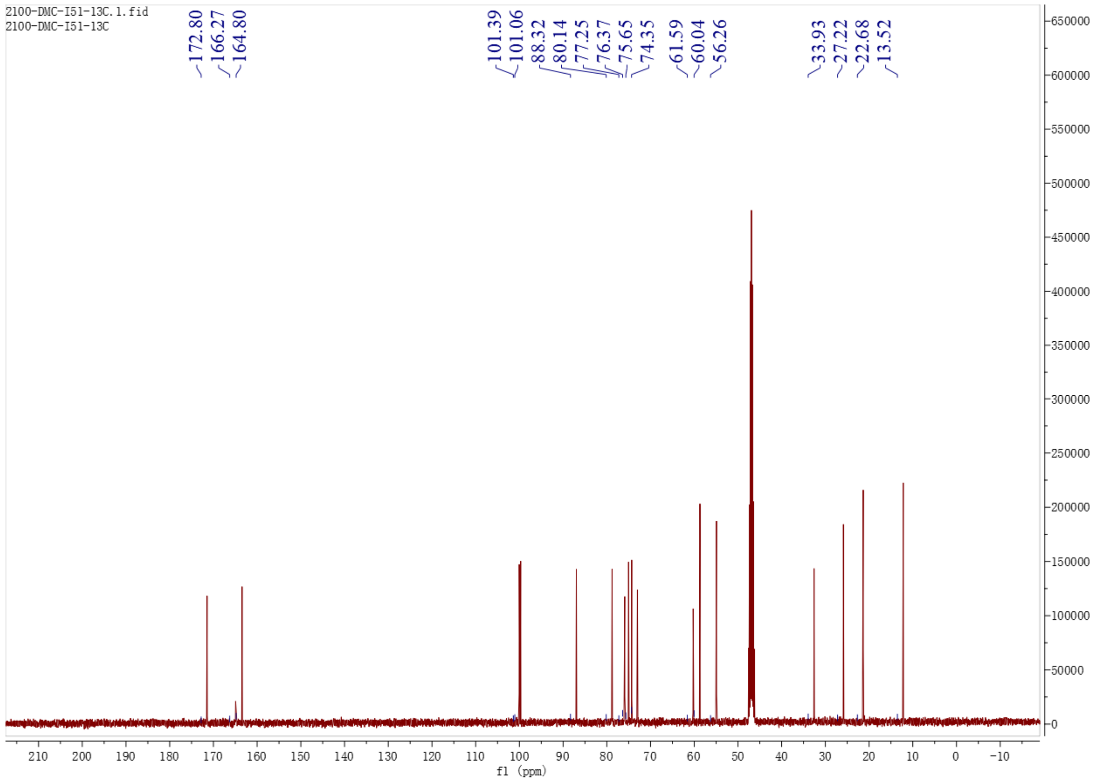


**Figure S17.** $^{13}$C NMR (100 MHz, Methanol-*d*4) spectrum of compound **3**

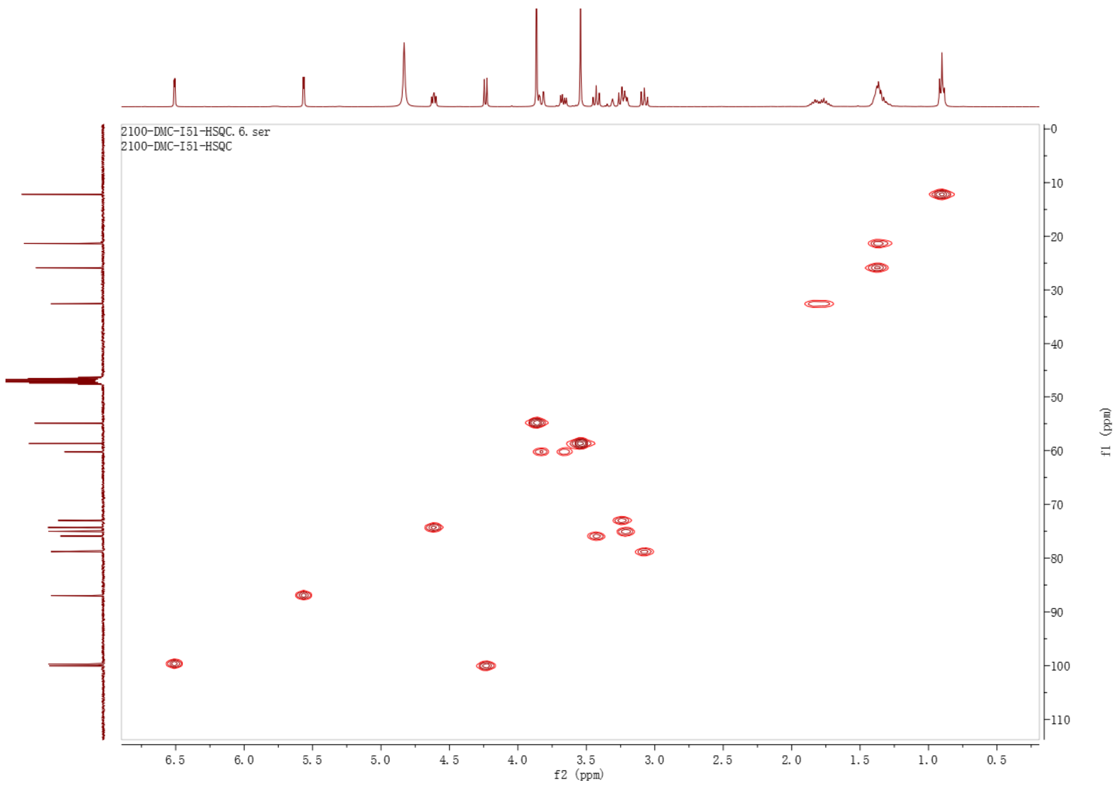


**Figure S18.** The HSQC (Methanol-*d*4) spectrum of compound **3**

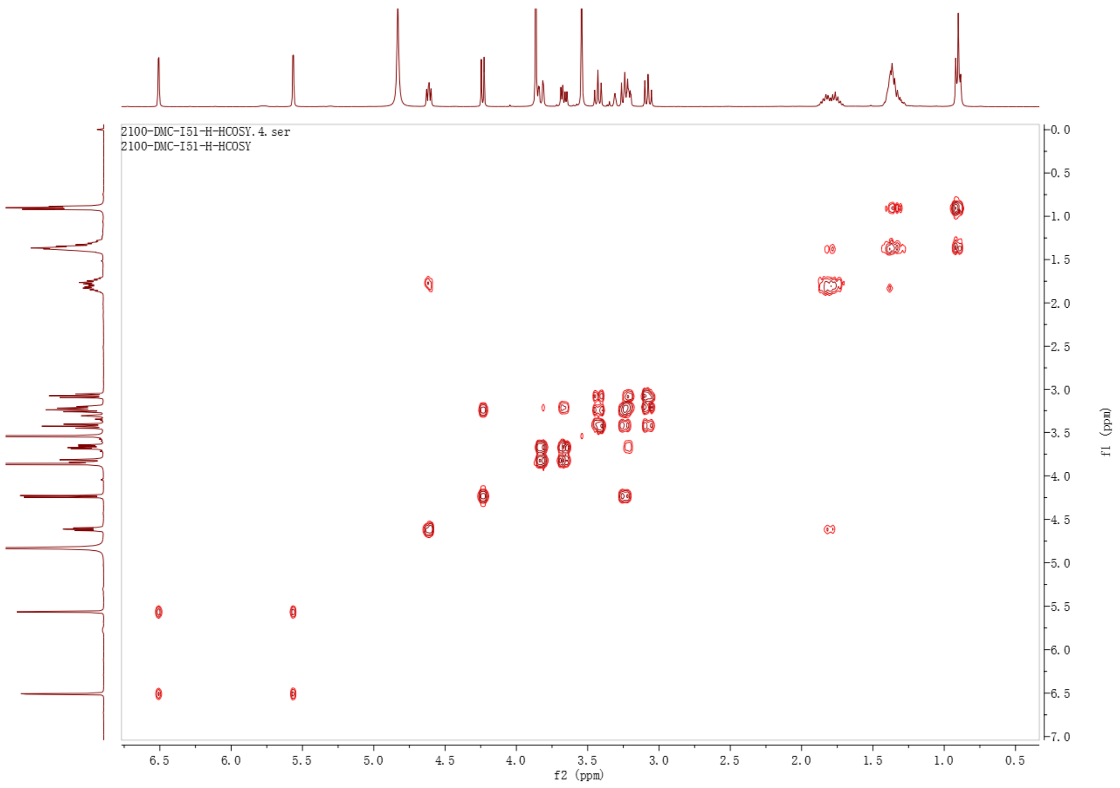


**Figure S19.** The $^1H$-$^1H$ COSY (Methanol-$d_4$) spectrum of compound **3**

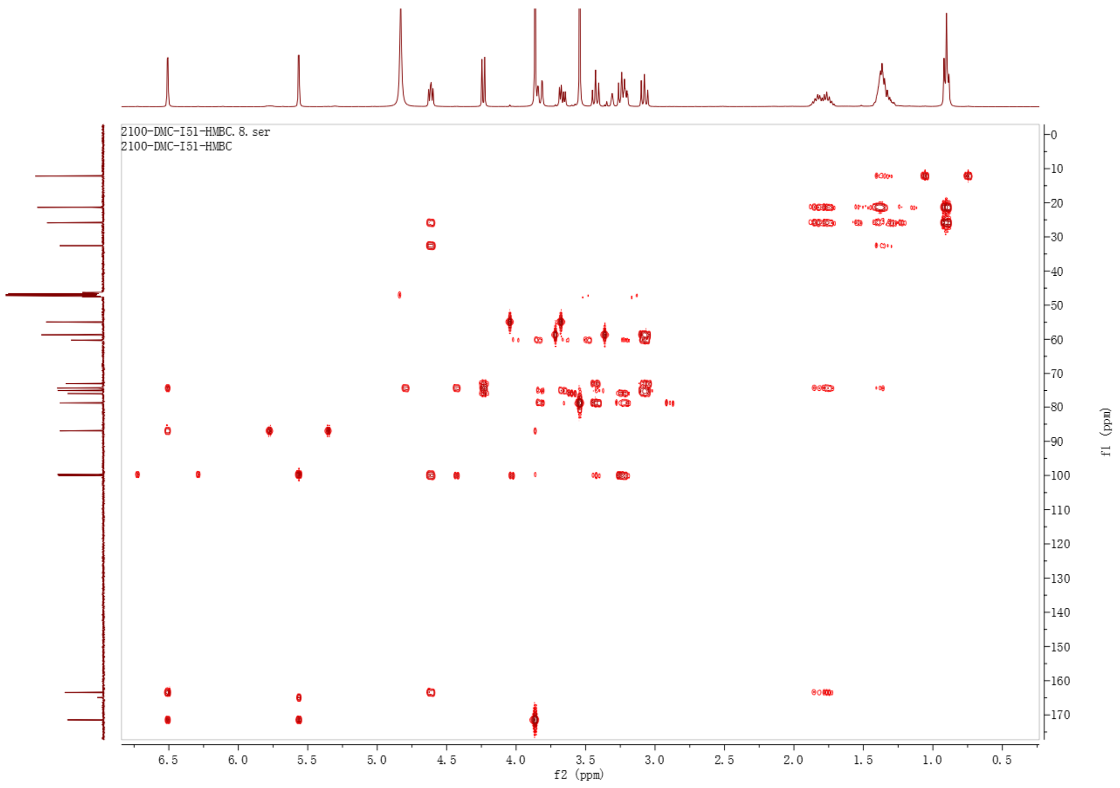


**Figure S20.** The HMBC (Methanol-$d_4$) spectrum of compound **3**

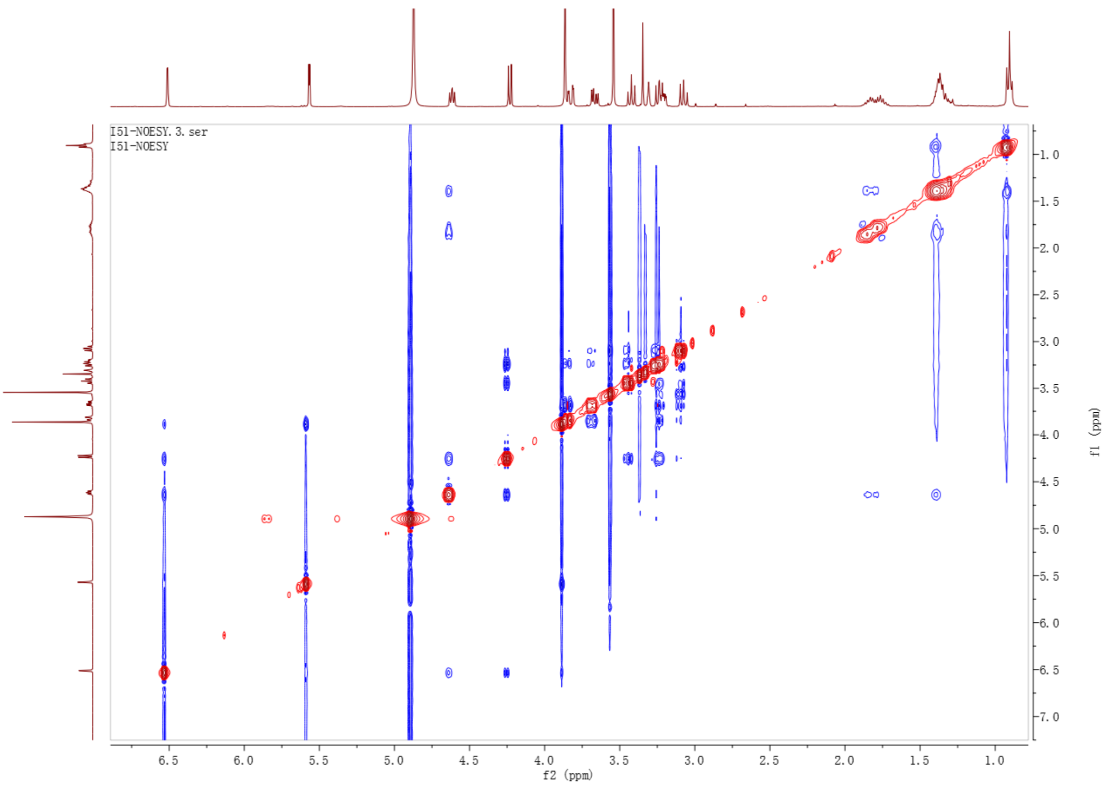


**Figure S21.** The NOESY (Methanol-$d_4$) spectrum of compound **3**

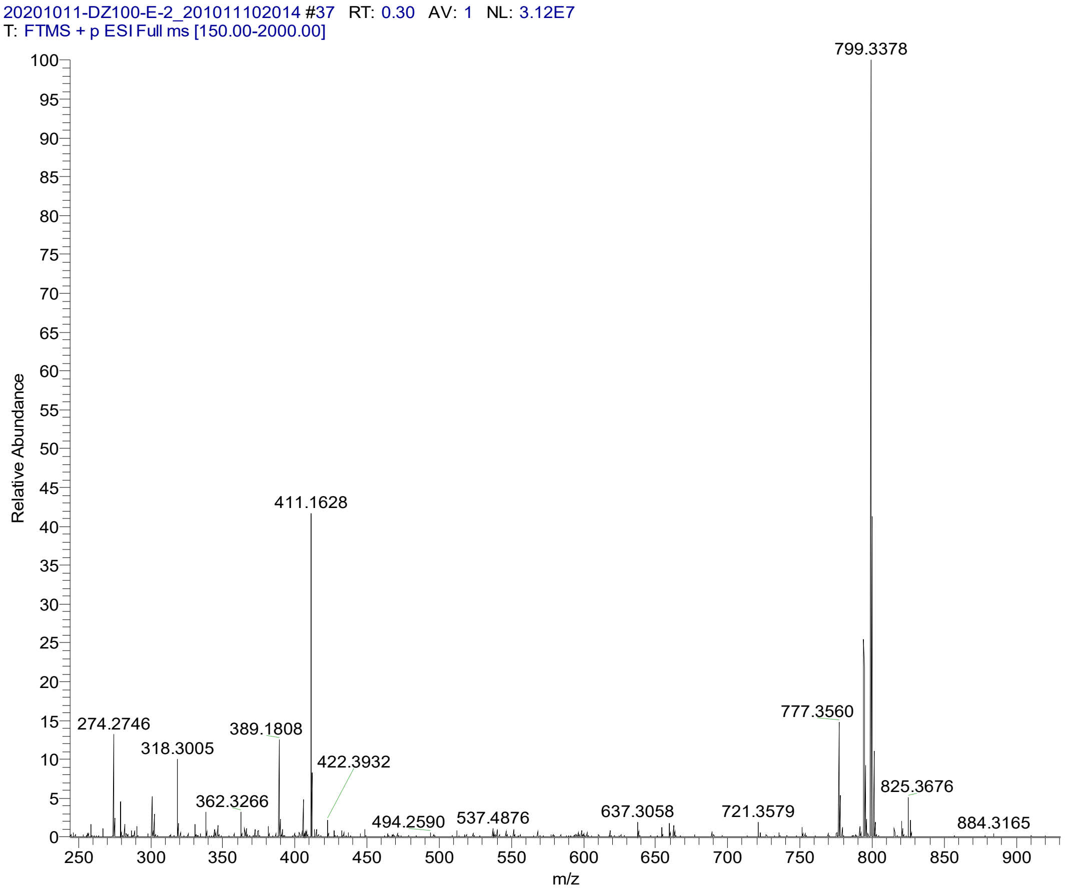


**Figure S22.** The HRESIMS spectrum of compound **3**

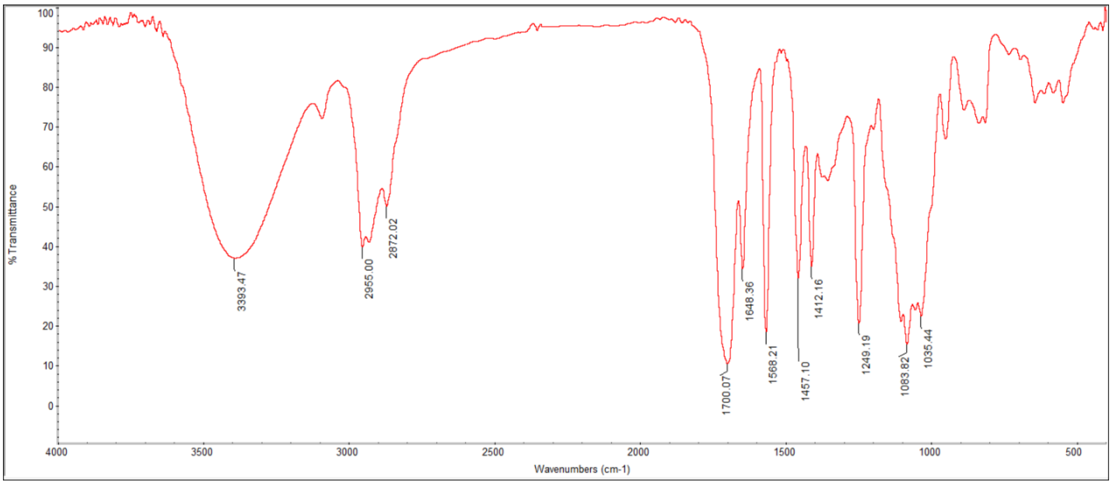


**Figure S23.** The IR spectrum of compound **3**

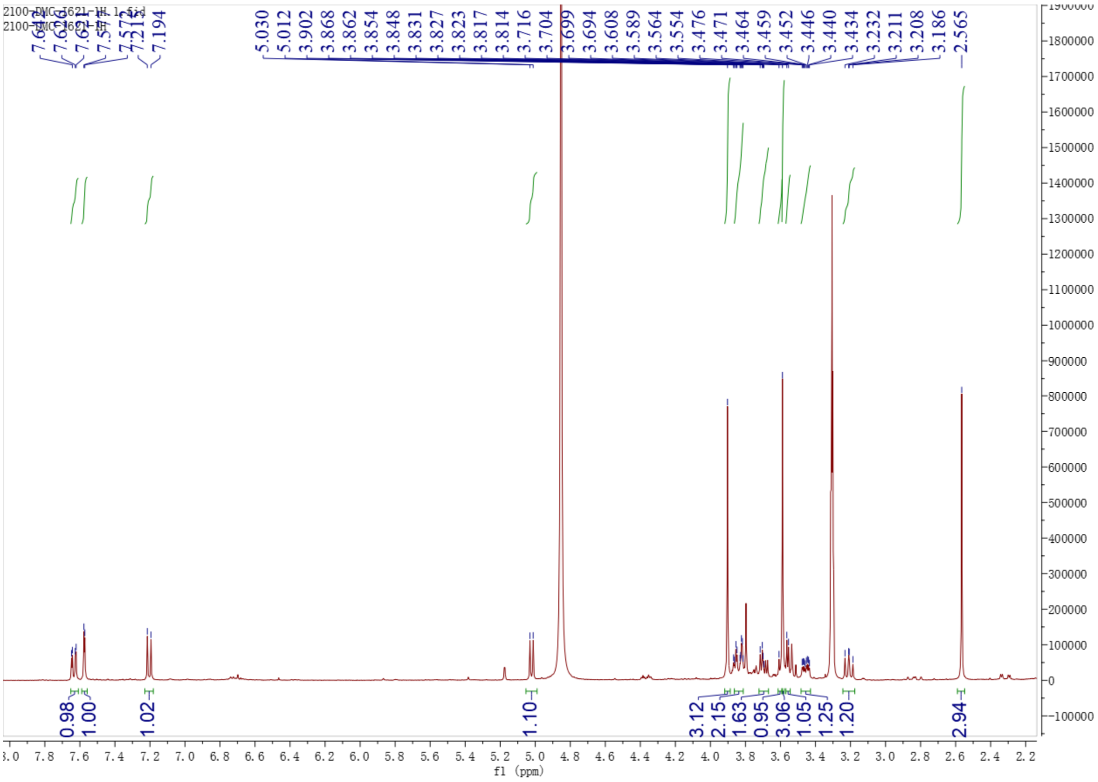


**Figure S24.** $^{1}$H NMR (400 MHz, Methanol-$d_4$) spectrum of compound **4**

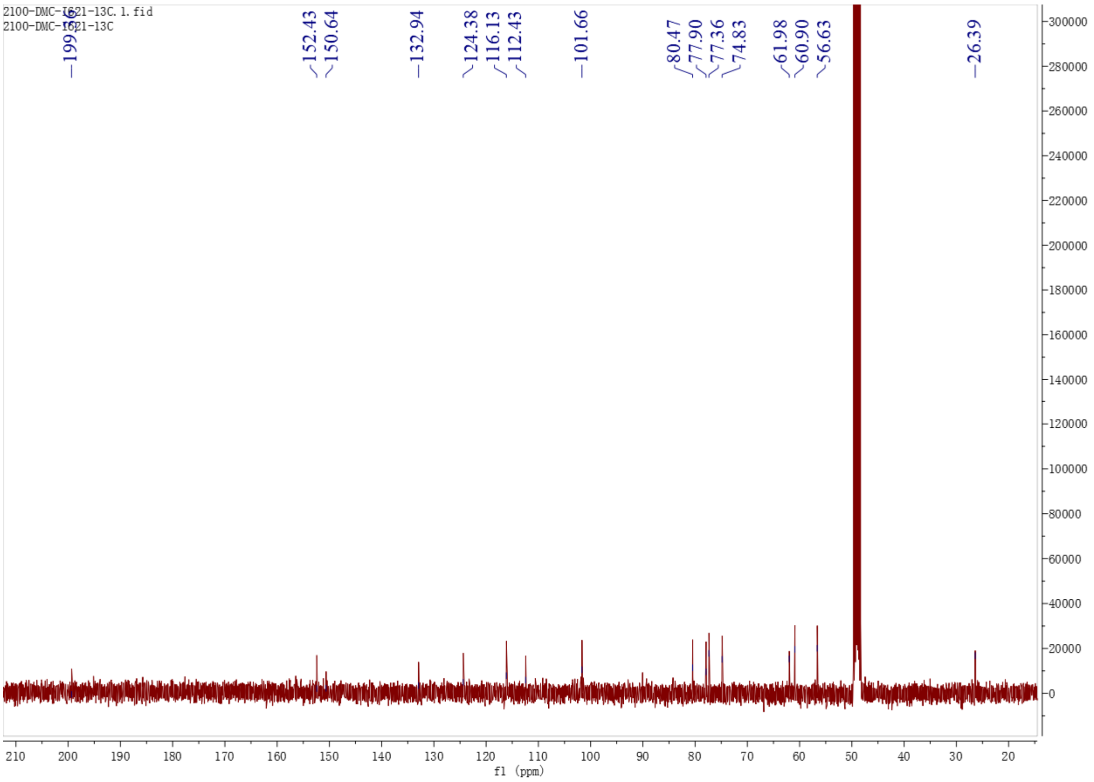


**Figure S25.** $^{13}$C NMR (100 MHz, Methanol-$d_4$) spectrum of compound **4**

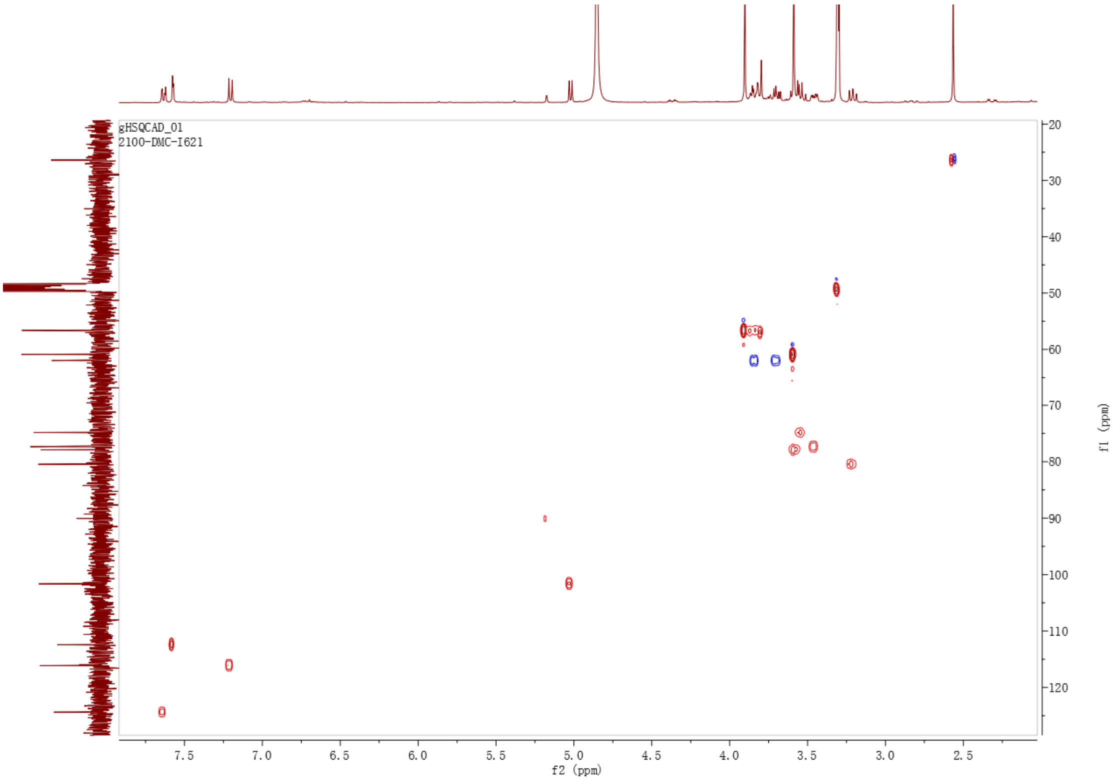


**Figure S26.** The HSQC (Methanol-$d_4$) spectrum of compound **4**

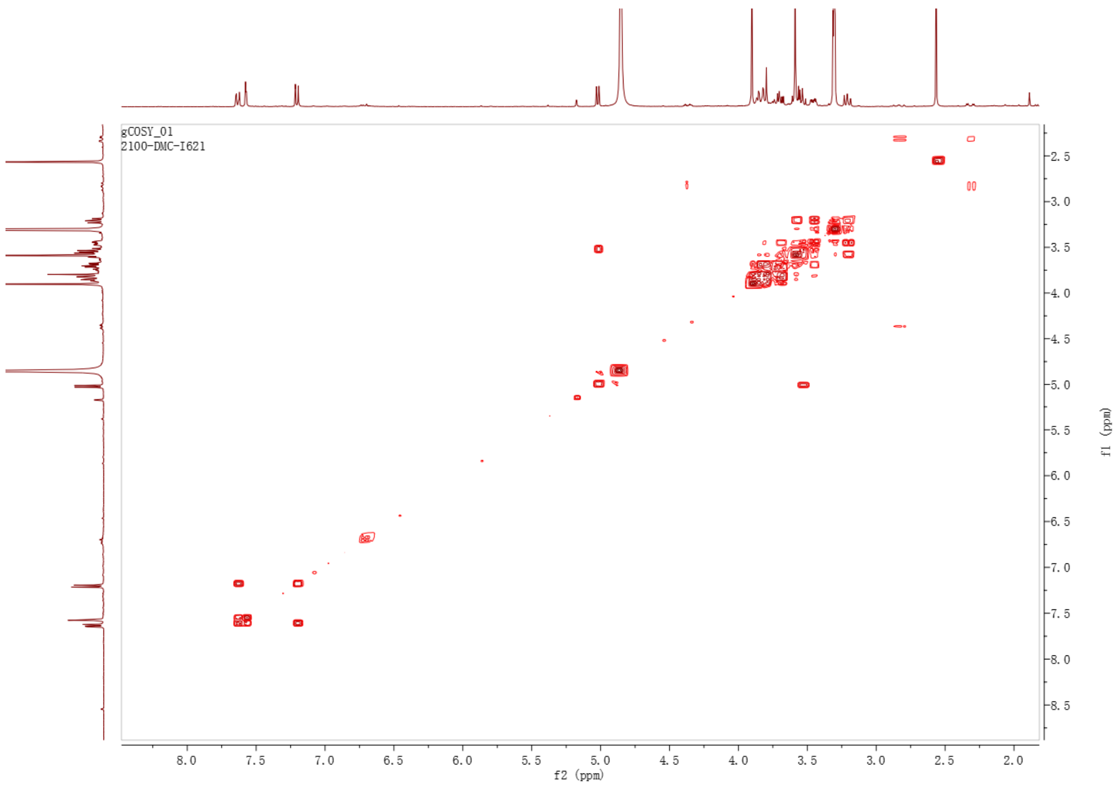


**Figure S27.** The $^{1}H$-$^{1}H$ COSY (Methanol-$d_4$) spectrum of compound **4**

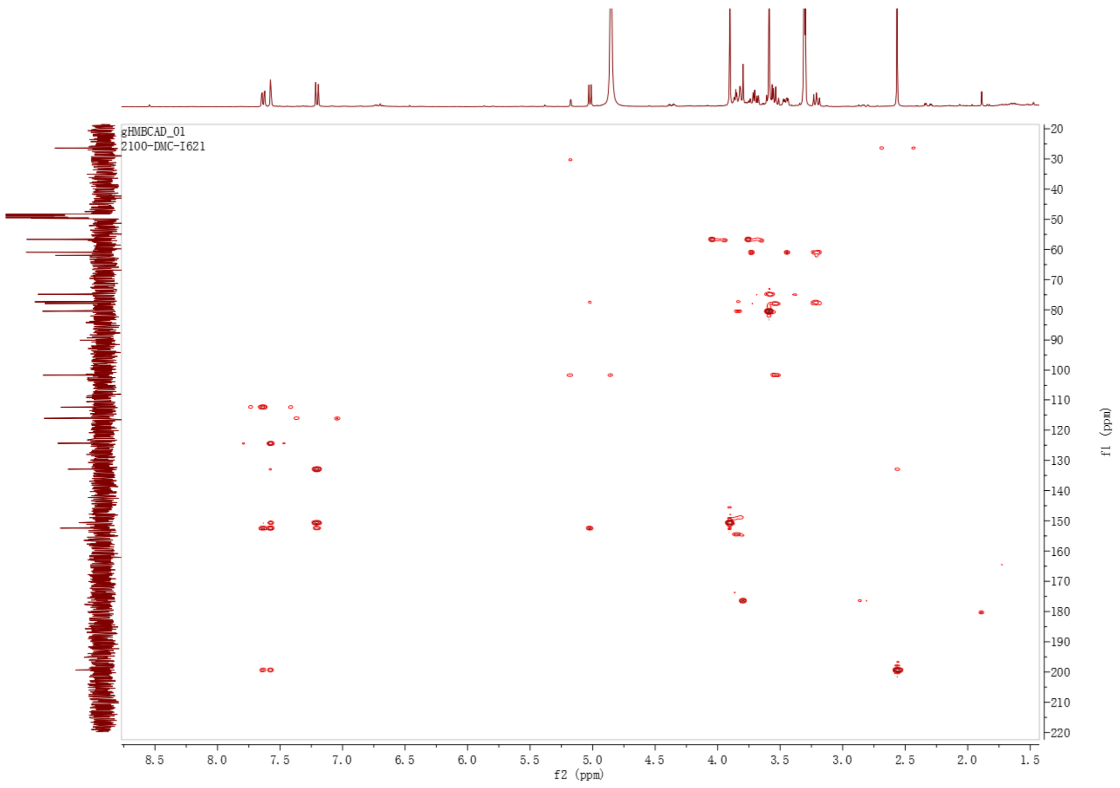


**Figure S28.**The HMBC (Methanol-$d_4$) spectrum of compound **4**

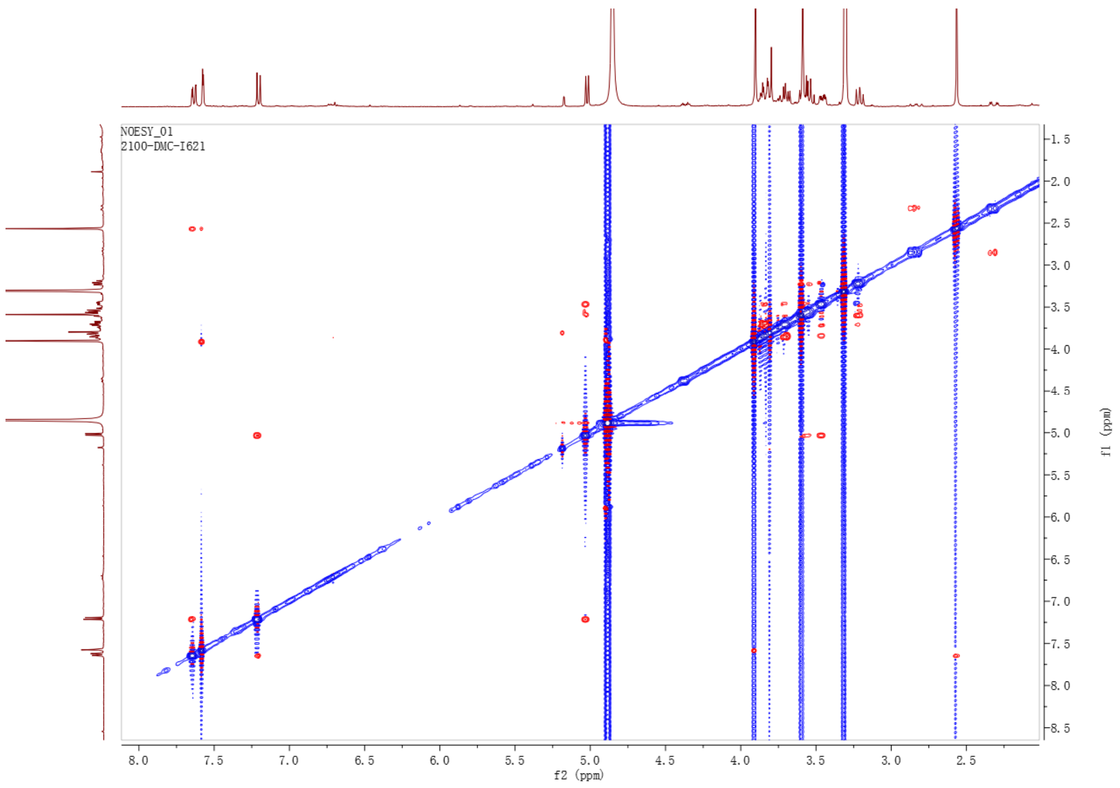


**Figure S29.** The NOESY (Methanol-$d_4$) spectrum of compound **4**

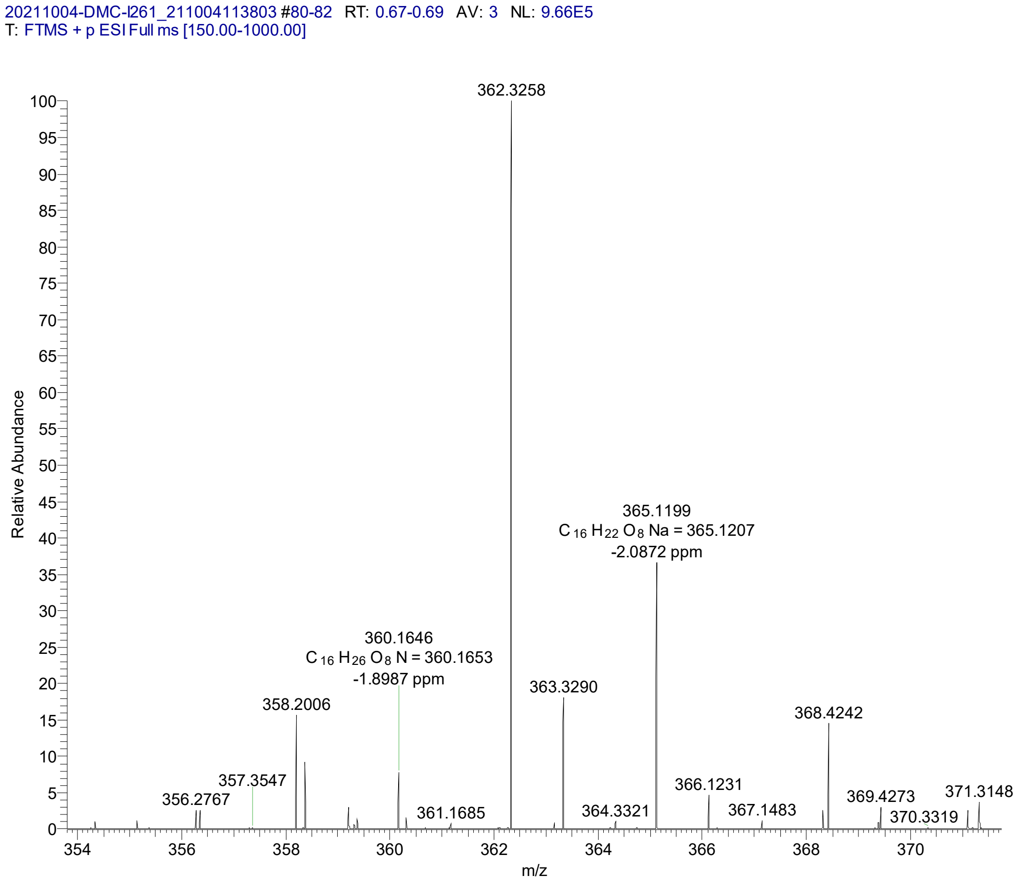


**Figure S30.** The HRESIMS spectrum of compound **4**

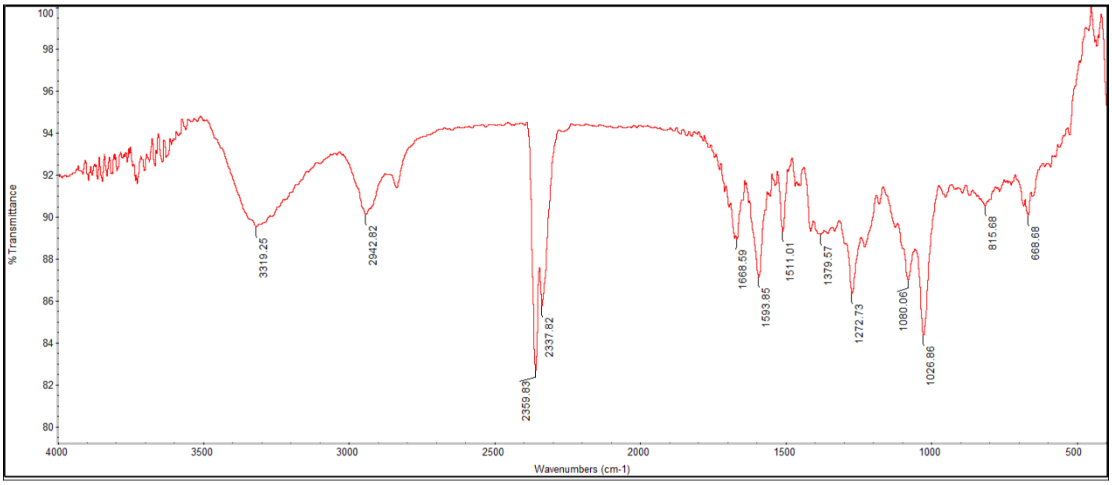


**Figure S31.** The IR spectrum of compound **4**
